# Microfibrillar-associated protein 5 suppresses adipogenesis by inhibiting essential coactivator of PPARγ

**DOI:** 10.1038/s41598-023-32868-y

**Published:** 2023-04-05

**Authors:** Tianlong Zhang, Haoran Li, Shiwei Sun, Wuling Zhou, Tieqi Zhang, Yueming Yu, Qiang Wang, Minghai Wang

**Affiliations:** 1 Department of Orthopedics, Shanghai Fifth People’s Hospital, Fudan University, No128. Ruili Road, Minhang District, Shanghai, 200240 China; 2 Department of Anatomy and Stem Cells and Metabolism Research Program, Faculty of Medicine, University of Helsinki, Helsinki, Finland; 3 Center of Community-Based Health Research, Fudan University, Shanghai, China

**Keywords:** Cell biology, Molecular biology, Stem cells

## Abstract

Femoral head necrosis is responsible for severe pain and its incidence is increasing. Abnormal adipogenic differentiation and fat cell hypertrophy of bone marrow mesenchymal stem cells increase intramedullary cavity pressure, leading to osteonecrosis. By analyzing gene expression before and after adipogenic differentiation, we found that Microfibril-Associated Protein 5 (MFAP5) is significantly down-regulated in adipogenesis whilst the mechanism of MFAP5 in regulating the differentiation of bone marrow mesenchymal stem cells is unknown. The purpose of this study was to clarify the role of MAFP5 in adipogenesis and therefore provide a theoretical basis for future therapeutic options of osteonecrosis. By knockdown or overexpression of MFAP5 in C3H10 and 3T3-L1 cells, we found that MFAP5 was significantly down-regulated as a key regulator of adipogenic differentiation, and identified the underlying downstream molecular mechanism. MFAP5 directly bound to and inhibited the expression of Staphylococcal Nuclease And Tudor Domain Containing 1, an essential coactivator of PPARγ, exerting an important regulatory role in adipogenesis.

## Introduction

Steroid-induced osteonecrosis of the femoral head (SONFH) is a common orthopedic disease with a high disability rate^1^. Long-term use of glucocorticoids induces necrosis of trabecular bone and bone marrow, eventually leading to hip dysfunction^2^. The pathogenesis of SONFH involves increased adipogenesis and fat cell hypertrophy in marrow, increasing intraosseous pressure and inducing avascular necrosis^3^. However, there are no effective methods to prevent and treat SONFH, which thus necessitates hip replacement, the main treatment of end-stage SONFH at the present time. However, hip replacement is costly and causes marked trauma. Reversing the disorders of lipid metabolism, such as inhibiting adipogenesis of bone marrow mesenchymal stem cells (BMSCs), might improve SONFH treatment.

Fat cells in bone marrow are mainly derived from BMSCs^4^. The differentiation and maturation of adipocytes is complex and involves multiple signaling pathways, such as Erk, Wnt, BMP, and hedgehog pathways^5–7^. These affect the transcriptional activity of key factors, such as peroxisome proliferator-activated receptor-g (PPARγ), and CCAAT/enhancer binding protein alpha (CEBPα), which promote the initiation and maturation of adipogenesis^8,9^. These mechanisms maintain normal adipogenic differentiation by complex mutual regulation. Among them, the PPARγ signaling pathway is important in adipogenic differentiation and maturation^10^. A hormonal adipogenic stimulus triggers expression of CCAAT/enhancer-binding proteins, inducing the expression of PPARγ and initiating a series of downstream adjustment processes^11^. Transcriptional activation of PPARγ and other nuclear hormone receptors regulates the participation of co-activators or co-repressors that link nuclear receptors with the basal transcription machinery. Previous studies indicated the special role of PPARγ in SONFH. Pravastatin was found to prevent SONFH by suppressing the expression of PPARγ and activating Wnt pathway at both the mRNA and protein levels^12,13^, and Huogu I formula has been specifically shown to inhibit fat formation-related gene expression and the occurrence of SONFH by inhibiting the expression of the PPARγ gene^14^. SND1 (Staphylococcal Nuclease And Tudor Domain Containing 1), also known as Tudor staphylococcal nuclease (Tudor-SN) or p100, is a co-activator of PPARγ, which, by directly binding PPARγ, promotes the transcription of downstream factors and adipogenic differentiation^15^.

MFAP5, also known as Microfibril-Associated Glycoprotein 2 (MAGP2), is a 25 kDa glycoprotein, present in the stroma and extracellular matrix of all tissues. MFAP5 secreted by mesenchymal stromal cells plays a key role in the hematopoietic and immune systems^16,17^. Mutation of MFAP5 is associated with the pathology of thoracic aortic aneurysm and dissection in human^18^. Moreover, MFAP5 regulates the progression of ovarian cancer, breast cancer, and tongue cancer^19–21^. Previously, our research group found that MFAP5 promoted osteogenic differentiation of MSCs by activating the Wnt/β-catenin and AMPK signaling pathways^22^. However, the function of MFAP5 in regulating the differentiation of BMSCs remains unclear. By analyzing the data of adipogenic precursor cells and mature adipocytes, we found that the expression of MFAP5 was significantly down-regulated, implicating MFAP5 in adipogenesis. We verified the sequencing results and confirmed the function of MFAP5 in adipogenesis by silencing and overexpressing. In addition, we validated the mechanism of its regulation of BMSC differentiation. MFAP5 directly bound to and inhibited the expression of SND1, a novel coactivator of peroxisome PPARγ, suppressing the expression of downstream molecules of PPARγ including CD36 and Adipsin^23,24^. In summary, MFAP5 is involved in the regulation of adipogenic differentiation, and has potential as a therapeutic target for diseases caused by adipogenic over-differentiation, such as SONFH.

## Materials and methods

### Microarray data

The gene expression profiles of undifferentiated and differentiated adipocytes, including GSE20696, GSE40565, and GSE119593, were acquired from the GEO database (https://www.ncbi.nlm.nih.gov/). The GEO2R web tool was used to identify differentially expressed genes with screening criteria of an adjusted *P* < 0.05 and an absolute logFC value of > 1.5 after eliminating invalid ​​and duplicate values. Next, the intersection of the differentially expressed genes was found and the top 30 (ranked by the mean of the absolute value of logFC) differentially expressed genes were visualized.

### Reagents and drug preparation

Dexamethasone (DXMS, D4902), L-Ascorbic acid (AA, A4403), β-Glycerophosphate (β-GP, G9422), Isobutylmethylxanthine (IBMX, I5879) and Indomethacin (ID, I7378) were purchased from Sigma Aldrich, USA. The antibody to MFAP5(DF13146) was from Affinity, USA. Antibodies to SND1(A5874), IgG (AC005), PPARγ(A19676), CD36(A19016), Adipsin (A8117), and β-actin(AC026) were from ABclonal, CHN. Goat anti-rabbit(7074) and -mouse(4410) antibodies from CST, USA were used as secondary antibodies.

### Cell culture and differentiation

The 293 T (for lentivirus packaging), 3T3-L1, and C3H10 cell lines were purchased from the Cell Bank of the Chinese Academy of Sciences, Shanghai, and placed in high-glucose DMEM containing 10% FBS; the medium was exchanged every 3 days. 3T3-L1 and C3H10T1/2 cells were induced to differentiate when they reached 100% confluence in differentiation medium, (500 mM IBMX, 200 mM indomethacin, 1 μM dexamethasone, and 10 μM insulin in growth medium). The differentiation medium was renewed every 2 days.

### Plasmids and viral infection

Three plasmids (psPAX2, pLKO.1-EGFP-puromycin, and pMD2.G) were purchased from GeneChem, China. Three shRNA sequences targeting mouse MFAP5 were designed for knockdown of MFAP5 expression (shRNA1: 5′-CCGGCGGGATGAGAAGTTTGCTTGTCTCGAGACAAGCAAACTTCTCATCCCGTTTTTTG-3′; shRNA2: 5′-CCGGGAGATGATGTGCCTGAGACATCTCGAGATGTCTCAGGCACATCATCTCTTTTTTG-3′; shRNA3: 5′-AAAACACCAGTTTACGACGTATGTATTCGTACATACGTCGTAAACTGGTGC-3′) and 3 to SND1 (shRNA1: 5′-CCGGGAAGGCATGAGAGCTAATAATCTCGAGATTATTAGCTCTCATGCCTTCTTTTTG-3′; shRNA2: 5′-CCGGTGTGGCTCCCACAGCTAATTTCTCGAGAAATTAGCTGTGGGAGCCACATTTTG-3′; shRNA3: 5′-CCGGTCTCGTCTCAAACTCAAACTCTATTTGCTCGAGCAAATAGAGTTTGAGACGAGATTTTG-3′).

The full-length coding sequence of mouse MFAP5 was amplified and inserted into a lentiviral vector CMV-MCS-EGFP-SV40-Puro to overexpress MFAP5 in C3H10 and 3T3-L1 cells. To produce lentiviruses, a lentiviral vector (pLKO.1-puro or pCDHCMV-MCS-EF1-Puro), psPAX2, pMD2.G, and Lipofectamine2000 (Invitrogen) were mixed and added to 293 T cells in high-glucose DMEM without FBS. The cell density was about 80%. At 12 h after transfection, the medium was changed to high-glucose DMEM with 10% FBS. Two days later, the supernatants were collected and filtered through a 0.45 μm membrane (Millipore). Cells were infected with lentivirus using 6 μg/mL polybrene. Next, puromycin was used to screen for stably transfected cells at a concentration of 3 μg/mL for 3 days and 1 μg/mL for 1 week.

### Oil red O staining

Oil red O staining was performed to assay lipid accumulation. Differentiated cells were rinsed with PBS three times and fixed in 4% formaldehyde for 25 min at 21 °C. Saturated Oil red O stain (0.5% in isopropanol) was diluted 60% with ddH2O and added to wells for 1 h at 21 °C. The cells were rinsed in 75% ethanol to remove residual Oil red O stain, and stained cells were observed and photographed.

### Quantitative real-time PCR

RNAiso Plus (9108, TaKaRa) was used to extract total RNA according to the manufacturer's instructions. RNA samples (500 ng) were reverse transcribed into cDNA with Primescript™ RT Master Mix (RR036A, TaKaRa). Quantitative real-time PCR reactions were implemented in a total volume of 10 μL, comprising 5 μL of 2 × SYBR Premix Ex Taq, 1 μL of diluted cDNA, and 0.2 μM primers. The amplification program was as follows: 95 °C for 10 min, followed by 40 cycles of 15 s at 95 °C and 34 s at 55 °C, then melting curve analysis for 15 s at 95 °C, 1 min at 55 °C, 15 s at 95 °C and 15 s at 60 °C. Each sample was established three holes and detected in three times independently. The 2^−ΔΔCT^ method was used to analyze the data. β-actin was set as the internal reference to normalize gene expression between samples. The primers used for qRT-PCR were from PrimerBank (https://pga.mgh.harvard.edu/primerbank/) and are listed in Supplemental File 1.

### Western blotting

Culture medium was removed, and adherent cells were washed twice with cold PBS. Next, 60 μL of RIPA (p0013b, Beyotime) buffer with 1% PMSF (st506, Beyotime) were added to 60 mm dishes and the cells were scraped into EP tubes. Lysates were vortexed for 10 s every 5 min for 30 min at 0 °C and centrifuged at 12,000 rpm at 4 °C for 10 min to harvest supernatant. A BCA kit (P0010, Beyotime) was used to determine the total protein concentration. Protein samples were subjected to SDS-gel electrophoresis and transferred to PVDF membranes (Millipore), which were blocked using 5% non-fat milk for 2 h at room temperature. According to the molecular weight of different proteins, the blots were cut from the membranes into a single strip, and put into a 15 ml centrifuge tube containing primary antibody for incubation. Following incubation with the primary antibodies at 4 °C overnight, the PVDF membranes were rinsed with TBST three times for 10 min each and probed with a goat anti-rabbit or -mouse secondary antibody (7074 and 4410, CST) at room temperature for 2 h, followed by detection using an ECL Kit (Share-bio, China). Images were analyzed using the Fluor Chem E system (Proteinsimple) and results were quantified using ImageJ software (https://imagej.nih.gov/ij/).

### Co-immunoprecipitation

Cells were harvested at the indicated time points during differentiation. After washing twice with cold PBS, 300 μL of RIPA (p0013d, Beyotime) buffer with 1% PMSF (st506, Beyotime) were added to a 100 mm dish. Next, 1 mL syringes were used to pump cells for 10 min. Next, 40 μL of Protein L Magnetic Beads (HY-K0205, MCE) were incubated with the corresponding antibody for 30 min at room temperature and washed four times in PBST. Protein samples and treated magnetic beads were incubated for 1 h at room temperature on a rotator. The magnetic beads were collected and rinsed four times in PBST, and eluted with 20ul loading buffer at 100 °C for 10 min.

### Statistical analysis

Statistical analysis was performed using GraphPad Prism 8.0 software for Windows. Numerical data are means ± SD from at least three replicates. The independent two-tailed Student’s *t*-test was used to compare two groups, and one-way ANOVA for more than two groups. *P* < 0.05 indicated a significant difference.

## Results

### MFAP5 was down-regulated during adipogenesis

**Figure 1:**
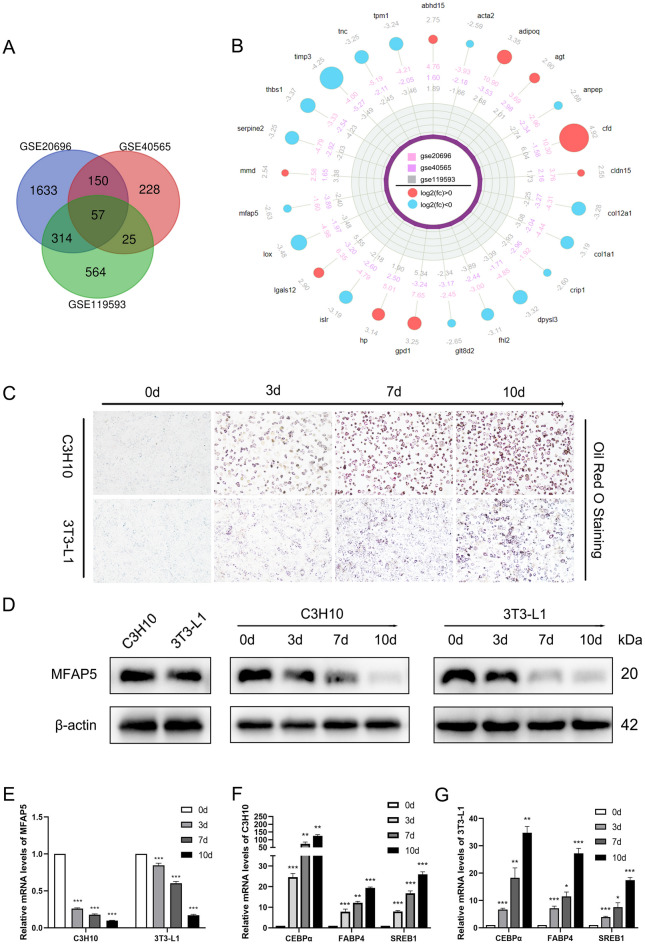
Association of MFAP5 with adipogenic differentiation. (**A**) Fifty-seven genes were at the intersection of three sequencing datasets before and after adipogenic differentiation. (**B**) Radar chart of 25 genes with the highest differential expression. Blue and red, down- and up-regulated during adipogenesis, respectively. Multiples of gene expression in each database are indicated by different colors. (**C**) Oil red O staining of C3H10 and 3T3-L1 cells. (**D**) Endogenous expression and expression pattern during adipogenesis of MFAP5 in C3H10 and 3T3-L1 cells. (**E**–**G**) Relative mRNA levels of MFAP5, CEBPα, FABP4, and SREB1 during adipogenesis. n = 3, the experiment was repeated 3 times. Values are means ± SD. *P < 0.05, **P < 0.01 and ***P < 0.001.

As shown in Fig. 1A, 57 differentially expressed genes were at the intersection of GSE20696, GSE40565, and GSE119593; the top 30 genes were shown in Fig. 1B. MFAP5 expression was consistently down-regulated. The adipogenic cell lines C3H10 and 3T3-L1 were used in adipogenic differentiation research^25–27^. Oil red O staining and analyzing the mRNA levels of CEBPα, FABP4, and SREB1 at 0, 3, 7, and 10 d of adipogenic induction showed that C3H10 and 3T3-L1 had good adipogenic differentiation potential (Fig. 1C, F and G). As shown in Fig. 1D and E, MFAP5 expression was stable in C3H10 and 3T3-L1 cells, and the MFAP5 protein and mRNA levels decreased gradually as adipogenic differentiation progressed, consistent with the bioinformatics data. The above results indicated a negative correlation between MFAP5 and adipogenic differentiation.

### Establishment of MFAP5-knockdown cell lines

**Figure 2:**
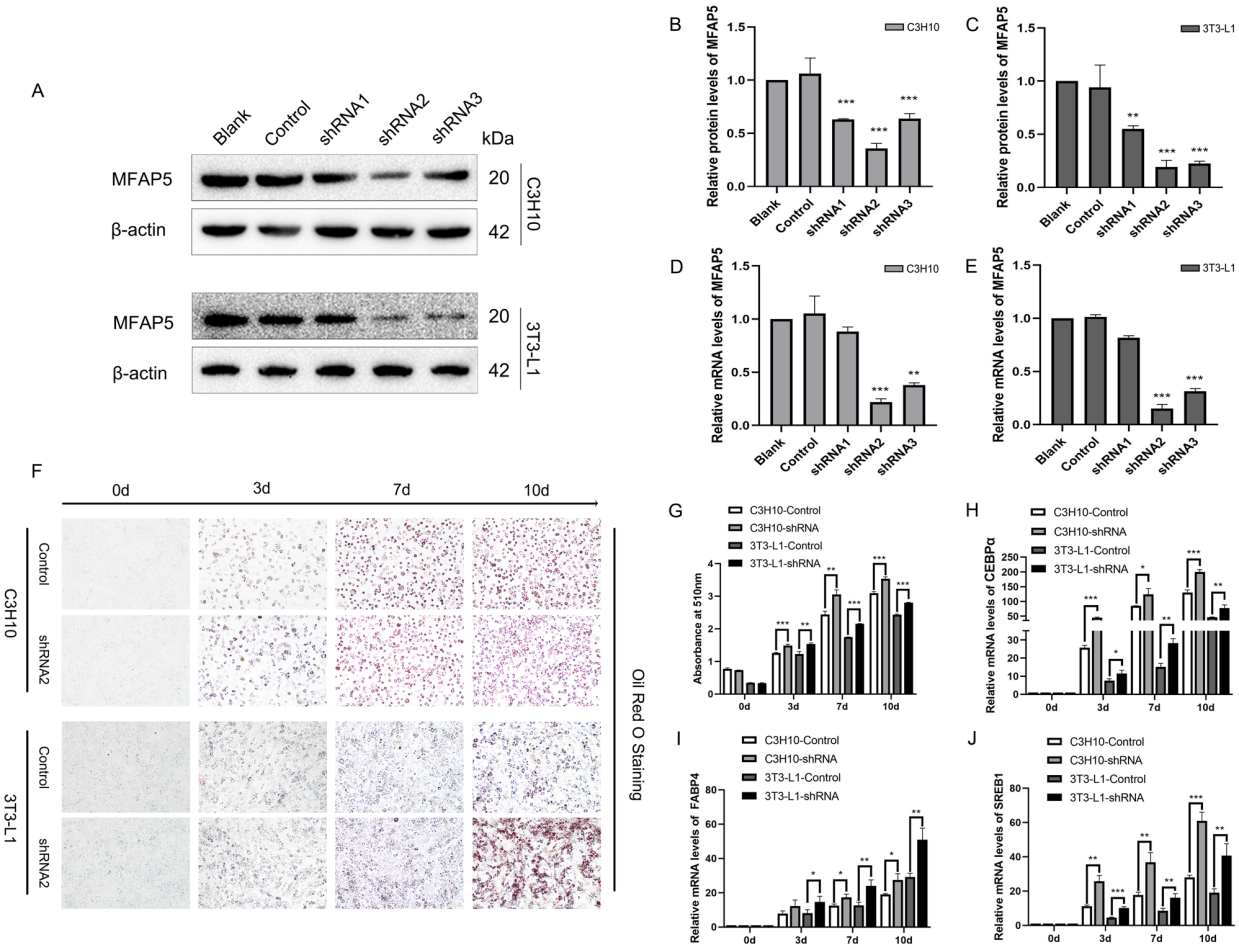
Establishment of MFAP5-knockdown C3H10 and 3T3-L1 cells and the role of MFAP5 in adipogenesis. (**A**) Western blotting of MFAP5 protein levels in the blank, control, and MFAP5-shRNA1-3 groups of C3H10 and 3T3-L1 cells. (**B**–**C**) Quantification of protein levels using ImageJ software in the blank, control, and MFAP5-shRNA1-3 groups of C3H10 and 3T3-L1 cells, respectively. (**D**–**E**) qRT-PCR of MFAP5 mRNA levels in the blank, control, and MFAP5 -shRNA1-3 groups of C3H10 and 3T3-L1 cells. (**F**) Lipid droplets accumulation determined by Oil red O staining. (**G**) Absorbance at 510 nm of Oil red O-stained cells. (**H**–**J**) Expression of CEBPα, FABP4, and SREB1 during adipogenesis was suppressed in MFAP5-knockdown cells. n = 3, the experiment was repeated 3 times. Values are means ± SD. *P < 0.05, **P < 0.01, and ***P < 0.001.

An siRNA was used to knockdown expression of MFAP5 in C3H10 and 3T3-L1 cells. To enhance knockdown, three shRNA sequences were designed and used in combination with a lentivirus transfection system to establish stable expression cell lines. Western blotting (Fig. 2A-C) and qRT-PCR (Fig. 2D and E) showed that MFAP5 expression was most significantly silenced in the MFAP5-shRNA2 group in both cell lines.

### MFAP5 knockdown promoted adipogenesis and expression of adipogenic biomarkers

We evaluated the role of MFAP5 in adipogenesis by Oil red O staining (Fig. 2F) and by measuring absorbance (Fig. 2G). MFAP5 knockdown facilitated differentiation of C3H10 and 3T3-L1 cells from day 3 onward, as indicated by the presence of more lipid accumulating cells. During adipogenic differentiation, the mRNA levels of CEBPα, FABP4, and SREB1 increased compared with the control (Fig. 2H-J), consistent with the results of Oil red O staining. Collectively, these findings suggested that knockdown of MFAP5 significantly promoted adipogenic differentiation.

### MFAP5 suppressed adipogenesis by inhibiting SND1, a coactivator of PPARγ

**Figure 3:**
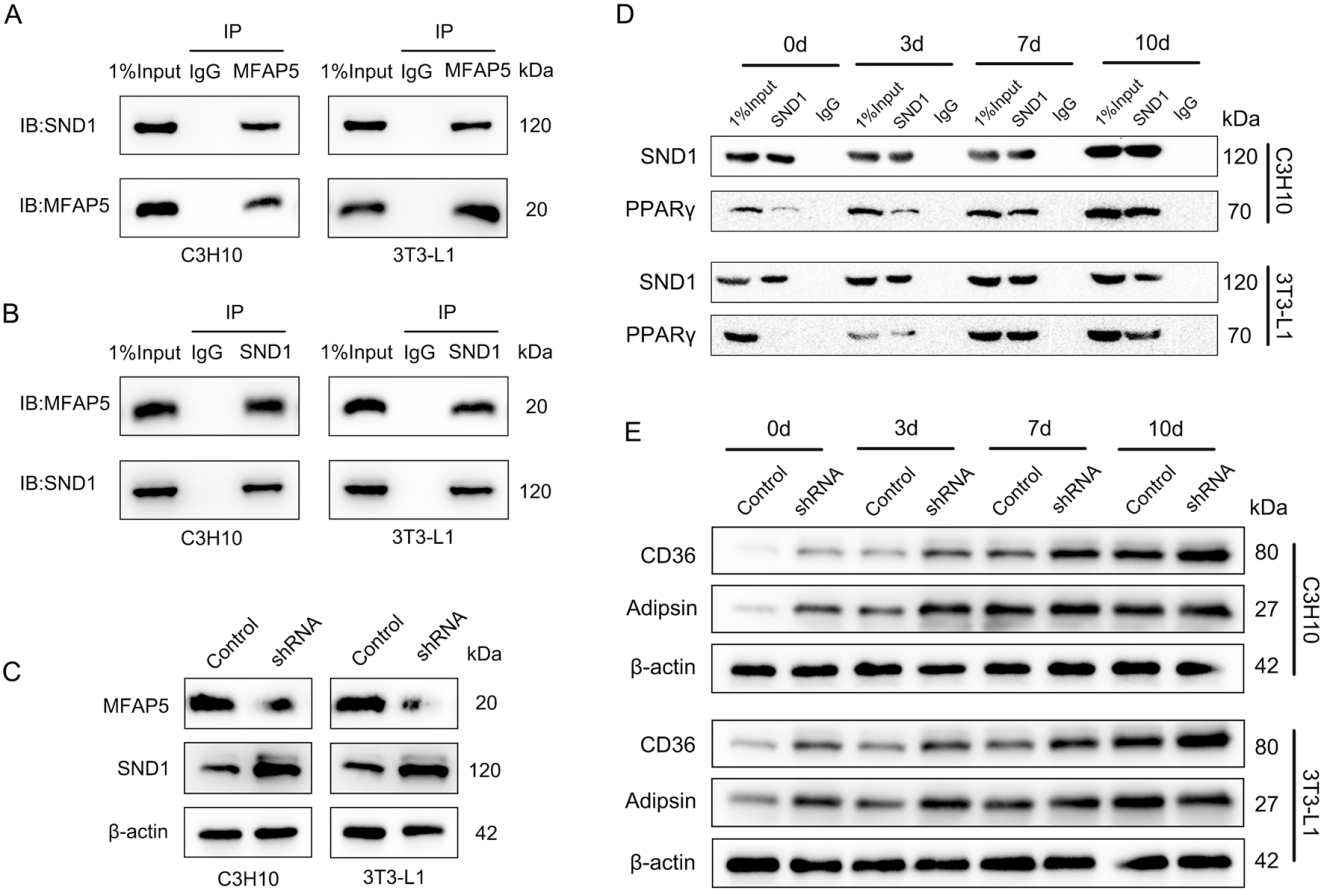
MFAP5 directly bound to and suppressed SND1, inhibiting activation of the PPARγ signaling pathway. (**A** and **B**) Co-immunoprecipitation showed MFAP5 and SND1 interacted at the protein level with an IgG antibody as a negative control in C3H10 and 3T3-L1 cells. (**C**) Knockdown of MFAP5 up-regulated SND1 expression. (**D**) During adipogenesis, SND1 expression was up-regulated and bound to PPARγ in C3H10 and 3T3-L1 cells. (**E**) CD36 and Adipsin (downstream genes of PPARγ) were significantly up-regulated in MFAP5-knockdown cells. n = 3, the experiment was repeated 3 times.

After ensuring the effect of MFAP5 on adipogenic differentiation, we further explored the underlying exact mechanism. Adipogenic differentiation involves complex pathway regulation. PPARγ requires auxiliary factors to regulate its downstream molecular transcription and trigger lipid accumulation, resulting in fat differentiation and maturation. Duan et al. reported that SND1 was an essential coactivator of PPARγ^15^. As shown in Fig. 3A and B, co-immunoprecipitation showed that MFAP5 directly bound SND1 in both cell lines. Furthermore, in MFAP5-knockdown cells, the expression of SND1 was significantly up-regulated (Fig. 3C). Therefore, MFAP5 directly bound to and inhibited the expression of SND1. To determine whether inhibition of SND1 mediated MFAP5-induced inhibition of adipogenic differentiation, we performed co-immunoprecipitation. The expression of SND1 increased upon adipogenic induction in C3H10 and 3T3-L1 cells (Fig. 3D). As adipogenic differentiation progressed, more PPARγ bound to SND1. During adipogenesis, the downstream proteins of PPARγ, including CD36 and Adipsin, were up-regulated in MFAP5-knockdown cell lines compared to the control (Fig. 3E). Therefore, MFAP5 directly binds to and suppresses SND1, inhibiting PPARγ-mediated transcriptional activation.

### Knockdown of SND1 reversed the inhibition of adipogenesis

**Figure 4:**
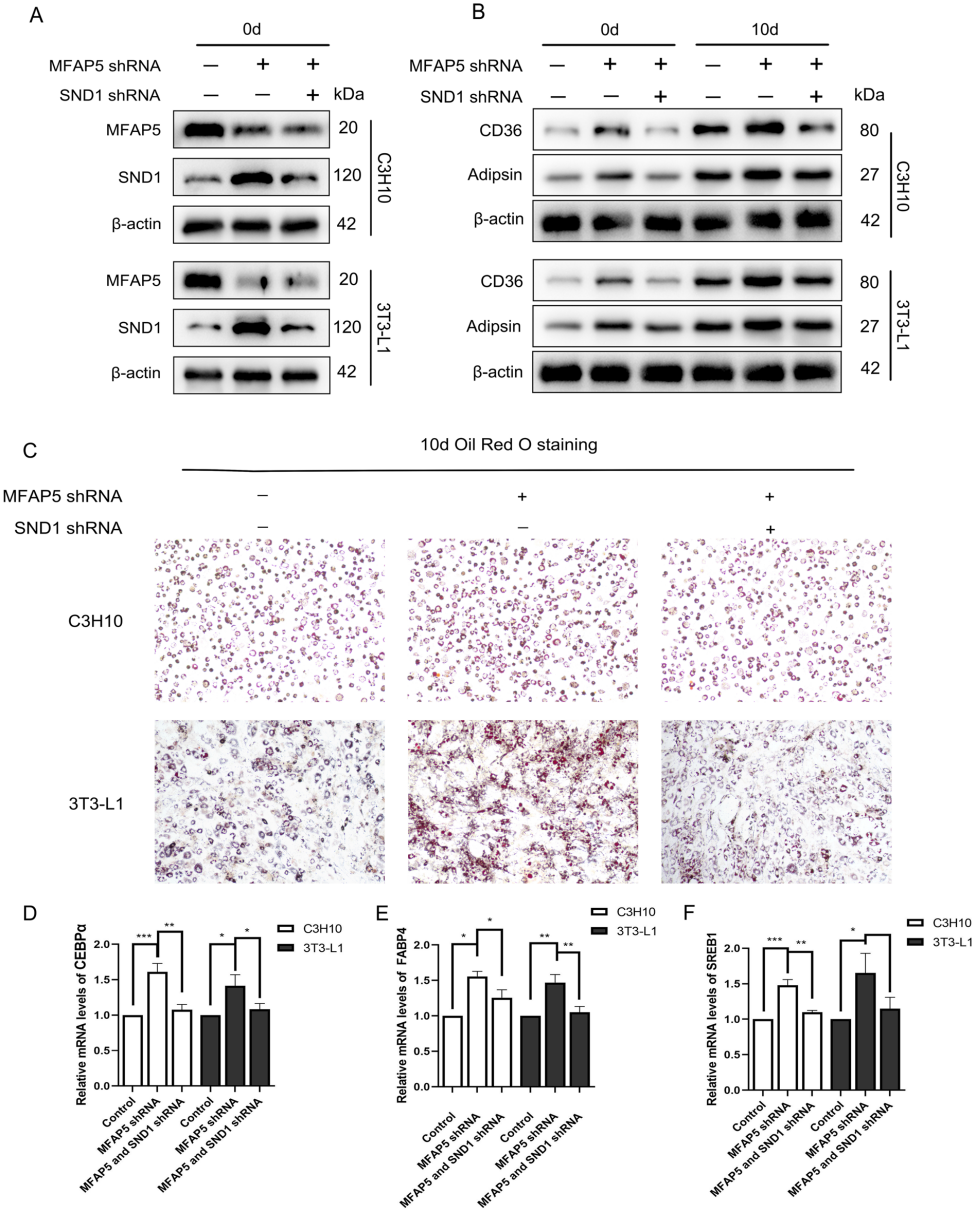
Silencing of SND1 in MFAP5-knockdown cell lines reversed the promotion of adipogenesis. (**A**) Western blotting of SND1 in MFAP5-knockdown cells compared to the control. (**B**) SND1 knockdown reversed the activation of PPARγ downstream proteins, including CD36 and Adipsin, in MFAP5-knockdown cells before and after adipogenic induction. (**C**) Accumulation of lipid droplets by Oil red O staining 10 days after adipogenic induction in the control, MFAP5-sh, and MFAP5-SND1-sh groups. (**D**–**F**) Relative expression levels of adipogenic biomarkers—CEBPα, FABP4, and SREB1—by qRT-PCR on day 10 of adipogenesis. n = 3, the experiment was repeated 3 times. Values are means ± SD. *P < 0.05, **P < 0.01 and ***P < 0.001.

To confirm that MFAP5 suppressed adipogenesis by inhibiting the expression of SND1, we knocked down SND1 in MFAP5-silenced cells and assessed their adipogenic differentiation capacity (knockdown efficiency is shown in Supplemental File 2). Compared to MFAP5-silenced cells, knockdown of SND1 relieved the overexpression of SND1 (Fig. 4A). At 0 and 10 d of adipogenic induction, downstream proteins (including CD36 and Adipsin) were reversed in MFAP5-knockdown cells, similar to the control group (Fig. 4B). Oil red O staining also indicated that knockdown of SND1 on the basis of MFAP5 silencing cell lines made its level of adipogenesis ability return to that of control group (Fig. 4C). We also analyzed the mRNA level of the adipogenic biomarkers CEBPα, FABP4, and SREB1 in three cell lines. As shown in Fig. 4D-F, knockdown of SND1 reversed the effect of MFAP5 silencing on adipogenic ability. Therefore, MFAP5 suppressed adipogenesis by inhibiting SND1, thereby restraining the PPARγ signaling pathway.

### MFAP5 overexpression inhibited adipogenesis by suppressing the expression of SND1

**Figure 5:**
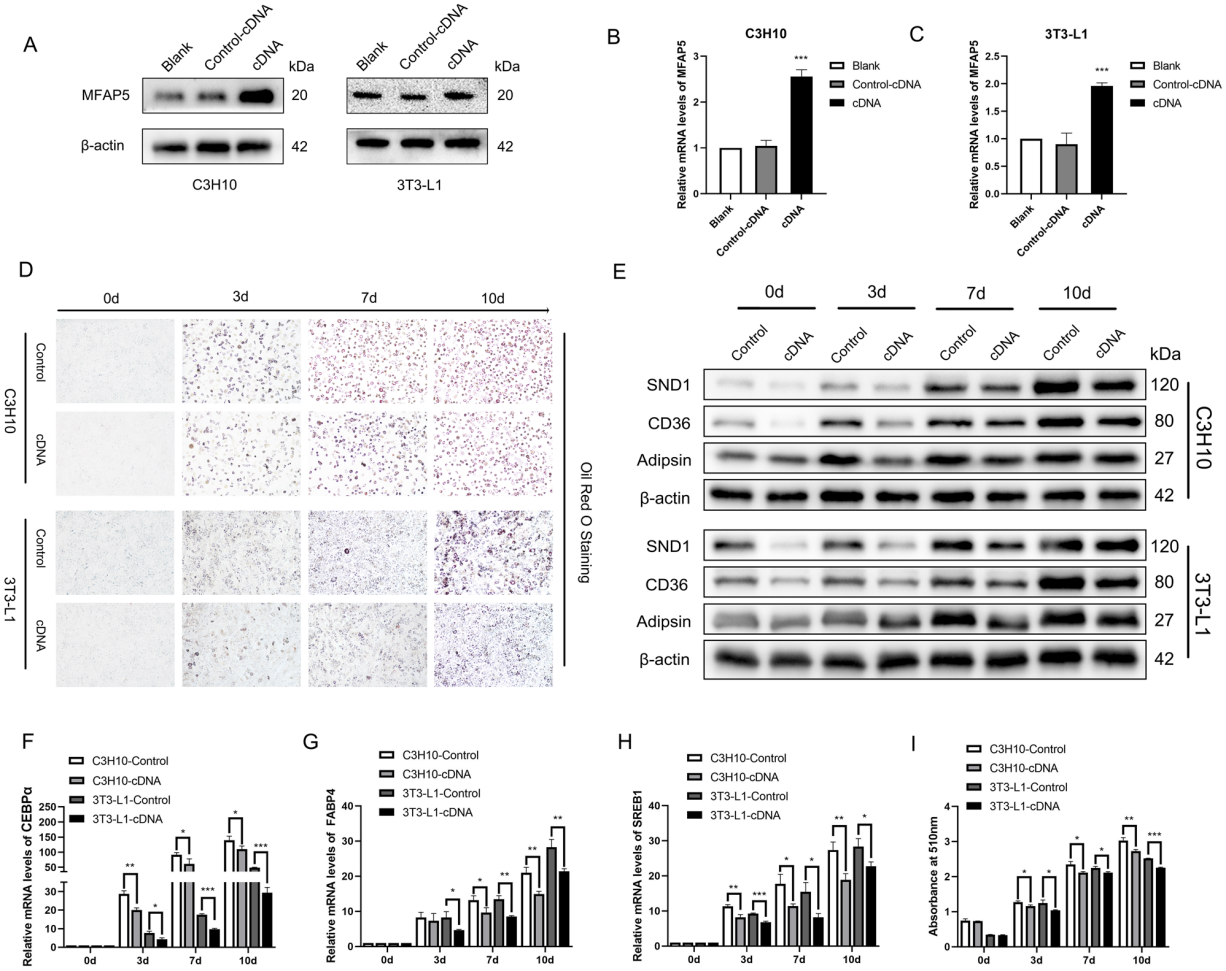
MFAP5 overexpression suppressed adipogenesis by inhibiting SND1. (**A**–**C**) Establishment of MFAP5-overexpressing C3H10 and 3T3-L1 cells. (**D**) Lipid droplets accumulation was significantly reduced in the MFAP5-overexpressing group. (**E**) Expression of SND1, CD36, and Adipsin during adipogenic induction in the control and MFAP5-overexpressing groups. (**F**–**H**). Relative mRNA levels of CEBPα, FABP4 and SREB1 showed that MFAP5 overexpression hindered adipogenic differentiation of C3H10 and 3T3-L1 cells. I. Absorbance at 510 nm of Oil red O-stained cells. n = 3, the experiment was repeated 3 times. Values are means ± SD. *P < 0.05, **P < 0.01 and ***P < 0.001.

To validate the above results, we established MFAP5 overexpression C3H10 and 3T3-L1 cell lines and evaluated their gene expression profiles (Fig. 5A-C). Oil red O staining (Fig. 5D) and absorbance measurement (Fig. 5I) showed that MFAP5 overexpression significantly inhibited adipogenic differentiation of both cell lines. This was supported by the mRNA expression levels of adipogenic biomarkers (Fig. 5F-H). At 0, 3, 7, and 10 d of adipogenic induction we found that overexpression of MFAP5 inhibited the expression of SND1. This suppressed CD36 and Adipsin, downstream proteins of PPARγ in adipogenesis (Fig. 5E). Therefore, MFAP5 negatively regulates adipogenic differentiation by directly binding to and inhibiting the expression of SND1, retarding the accumulation of lipid droplets and the maturation of fat cells.

## Discussion

Normal adipogenesis or adipocyte differentiation, which is regulated by a cascade of sequentially acting chromatin-modifying coregulators and transcription factors, plays an important role in balancing cell ratios in bone marrow. In older adults, the differentiation of adipocytes is enhanced, causing increased intraosseous pressure, avascular necrosis, and inhibition of homologous cell differentiation^28^. Here, we found that the expression of MFAP5 was significantly down-regulated during adipocyte differentiation and maturation. We verified the sequencing results and demonstrated the function of MFAP5 in adipogenesis by silencing or overexpression in C3H10 and 3T3-L1 cells^29–32^. As mesenchymal stem cells, C3H10 cells are more primitive than 3T3-L1 cells and have multidirectional differentiation ability. Both cell lines are used in adipogenic differentiation research. Our findings showed that MFAP5 inhibits adipogenesis by suppressing an essential coactivator of PPARγ.

MFAP5 is a component of extracellular elastic microfibrils, implicated in cardiovascular progression, breast cancer, carcinogenesis, and alveolar elastogenesis^18,20,33,34^. Maija et al.^35^ reported that MFAP5 was highly expressed in adipose tissue, and the expression of MFAP5 decreased during adipocyte differentiation in SGBS cells. However, they focused on adipose tissue inflammation and did not conduct an in-depth study of changes in gene expression or the role of MFAP5 in regulating adipocyte differentiation. During tumor development, MFAP5 may participate in the notch1, notch2, and Akt signaling pathways, which are related to adipocyte differentiation^36–38^. Therefore, MFAP5 may be involved in the regulation of adipogenesis.

The ligand-activated transcription factor PPAR, a nuclear receptor of the steroid, thyroid, and retinoic acid receptor superfamily, is the master regulator of adipogenesis^39^. After binding to ligands, activated PPAR combines with 9-cis-retinoic acid retinoid X receptors to form a heterodimer, then binds to the peroxisome proliferator response element of a target gene, activating its transcription^40^. PPARs play an important regulatory role in physiological processes such as fat synthesis, lipid metabolism, insulin sensitivity, and particularly the synthesis of enzymes involved in fatty acid β-oxidation^41^. According to their structure and function, PPARs are divided into three subtypes: PPARα, PPARβ/δ, and PPARγ^42^. Based on their promoter structure and mRNA splicing mode, PPARγ genes can be divided into PPARγ1, PPARγ2, PPARγ3, and PPARγ4. Among them, PPARγ1, PPARγ3, and PPARγ4 encode the same protein^43^. Compared with other types of PPARs, PPARγ is the most adipocyte-specific; its expression is high in adipose tissue and adipose cell lines, but low in other tissues and cell lines^44^. Activated PPARγ regulates the expression of adipocyte-related genes and promotes the differentiation and increases the number of adipocytes. Co-activators—including SND1, SRC-1, PRIP, p300 and TATA—are necessary for transcriptional activation of target genes^45–47^. Adipocyte differentiation is inhibited in the absence of these co-activators^15,47,48^. Duan et al.^15^ knocked out the expression of SND1 in 3T3-L1 cells and cultivated them in adipogenic induction medium for 8 days. Adipogenic differentiation almost completely stopped compared to control cells.

MFAP5 directly binds to SND1, as determined by co-immunoprecipitation, suggesting that the two proteins interact. In MFAP5-knockdown cells, the expression of SND1 was significantly inhibited. Notably, the downstream genes of PPARγ were markedly inhibited during adipogenesis in MFAP5-knockdown C3H10 and 3T3-L1 cells. Next, we knocked down the expression of SND1 in MFAP5-silenced cells, followed by adipogenic induction. As expected, the promotion of adipocyte differentiation by MFAP5-silenced cells was reversed. Therefore, SND1 is implicated in the negative regulation by MFAP5 of adipogenic differentiation.

## Conclusions

MFAP5 directly binds to and inhibits the expression of SND1. Our findings expand the upstream molecules of the PPARγ signaling pathway and suggest molecular targets for related research. MFAP5 knockdown may facilitate the development of novel therapeutic strategies for diseases caused by excessive adipogenic differentiation by inhibiting adipogenic differentiation of BMSCs in the femoral head of patients on long-term glucocorticoids.

## Supplementary Information

Supplementary Information.

## Data Availability

The gene expression profiles of undifferentiated and differentiated adipocytes, including GSE20696, GSE40565, and GSE119593, were acquired from the GEO database (https://www.ncbi.nlm.nih.gov/). The data that supported the findings of this study were available from the corresponding author upon reasonable request.

## Abbreviations

MFAP5: Microfibril associated protein 5
BMSCs: Bone marrow stromal cells
SND1: Staphylococcal nuclease and tudor domain containing 1
PPARγ: Peroxisome proliferator activated receptor gamma
SONFH: Steroid-induced osteonecrosis of the femoral head
CEBPα: CCAAT enhancer binding protein alpha
FABP4: Fatty acid binding protein 4
SREB1: G protein-coupled receptor 27
IBMX: Isobutylmethylxanthine
DXMS: Dexamethasone
ID: Indomethacin
DMEM: Dulbecco’s modified eagle medium
FBS: Fetal bovine serum
PBS: Phosphate buffered saline
PBST: Phosphate buffered saline tween
shRNA: Short hairpin RNA
qRT-PCR: Quantitative real-time PCR
PMSF: Phenylmethanesulfonyl fluoride
PVDF: Polyvinylidene fluoride
TBST: Tris buffered saline tween
