# Supplementary material for: Microfibrillar-associated protein 5 suppresses adipogenesis by inhibiting essential coactivator of PPARγ
Source: Sci Rep. 2023 Apr 5;13:5589. doi: 10.1038/s41598-023-32868-y (PMC10076305; doi:10.1038/s41598-023-32868-y)

Supplementary Information Files

Title

Microfibrillar-Associated Protein 5 Suppresses Adipogenesis by Inhibiting Essential Coactivator of PPARγ

Authors

Tianlong Zhang*[1,3], Haoran Li*[1,2], Shiwei Sun[1,3], Wuling Zhou[1,3], Tieqi Zhang[1,3], Yueming Yu[1,3], Qiang Wang[#1,3], Minghai Wang[#1,3]

*These authors contributed equally as first author to this work.

[#] Corresponding Authors.

Affiliations

1. Department of Orthopedics, Shanghai Fifth People's Hospital, Fudan University, Shanghai, China
2. Department of Anatomy and Stem Cells and Metabolism Research Program, Faculty of Medicine, University of Helsinki, Helsinki, Finland
3. Center of Community-Based Health Research, Fudan University, Shanghai, China

#Correspondence to:

Minghai Wang, Department of Orthopedics, Shanghai Fifth People's Hospital, Fudan University, No128. Ruili Road, Minhang District, Shanghai, China 200240. Phone: (+86)18021006917 E-mail: wangminghai@5thhospital.com.

#Co-Correspondence to:

Qiang Wang, Department of Orthopedics, Shanghai Fifth People's Hospital, Fudan University, No128. Ruili Road, Minhang District, Shanghai, China 200240. E-mail: wqwyj81@163.com.

Contents of Supplementary Information Files:

1. Supplemental File1: Primer sequences used for qRT-PCR
2. Supplemental File2: Knockdown efficiency of SND1
3. Supplemental File3: Original, unprocessed images of gels/blots used in this paper

Supplemental File 1

Primer sequences used for qRT-PCR

Gene	Forward	Reverse
mfap5	5’-CAGTCCTGCTTCACCAGTTTAC-3’	5’-AAGTCGGAAGTAGTTGGAGCG-3’
CEBPα	5’- GCGGGAACGCAACAACATC -3’	5’-GTCACTGGTCAACTCCAGCAC-3’
FABP4	5’-TCACCGCAGACGACAGGAGG -3’	5’-CACCACCAGCTTGTCACCATCTC-3’
SREB1	5’- TGACCCGGCTATTCCGTGA -3’	5’-CTGGGCTGAGCAATACAGTTC-3’
β-actin	5’- GGGACCTGACTGACTACCTC-3’	5’- TCATACTCCTGCTTGCTGAT-3’

Supplemental File 2

Knockdown efficiency of SND1

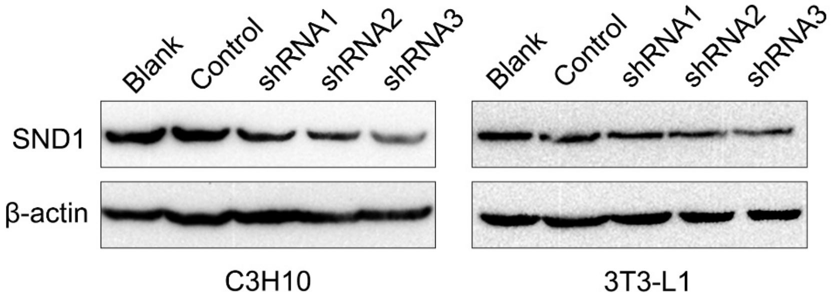

Knockdown efficiency of the three shRNA sequences used for knocking down the expression of SND1 in C3H10 and 3T3-L1 blank cells. We choose shRNA3 for further research in this paper.

Supplemental File 3

Original Images supporting Figure 1D:

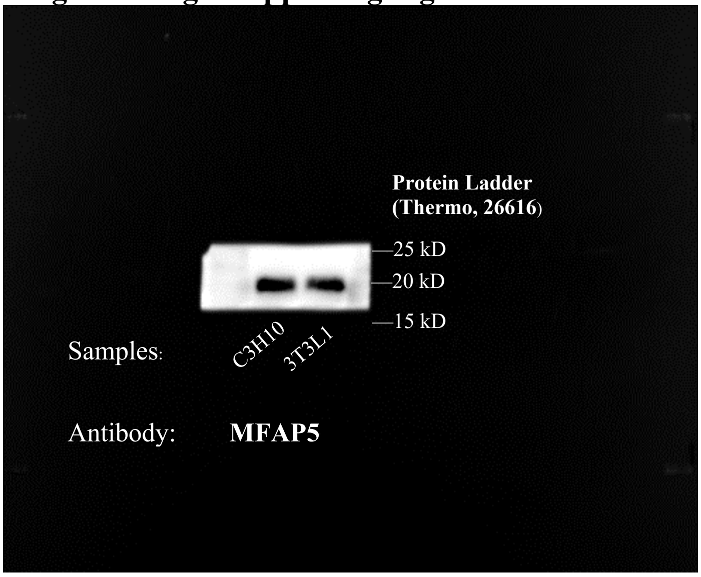

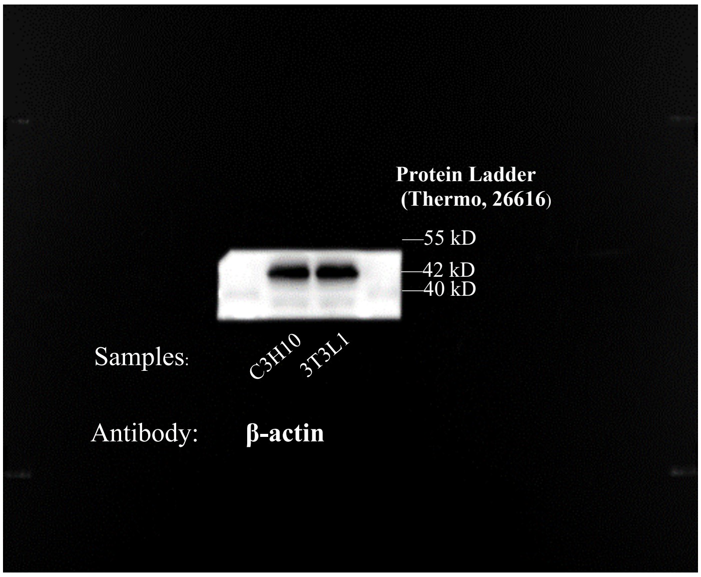

Cropped Figure 1D:

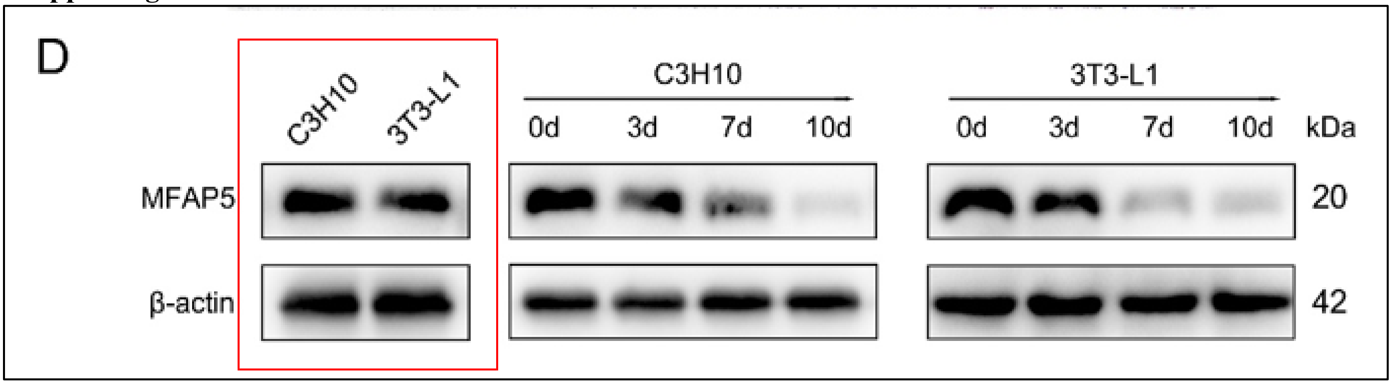

Original Images supporting Figure 1D:

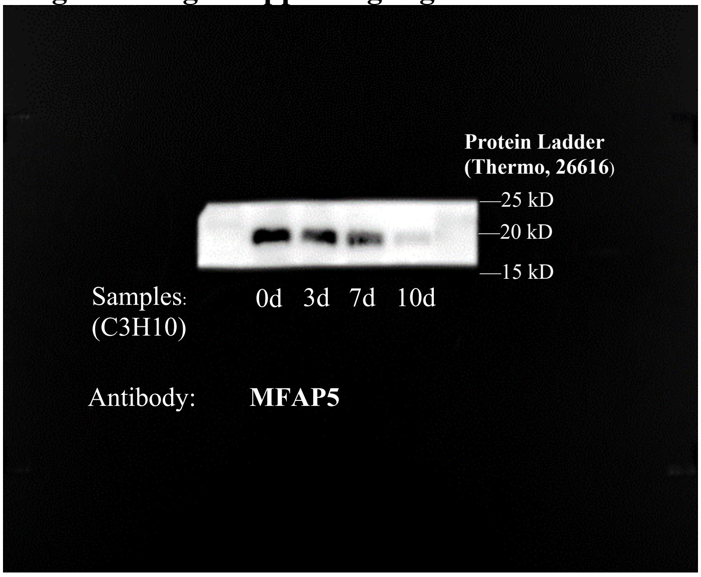

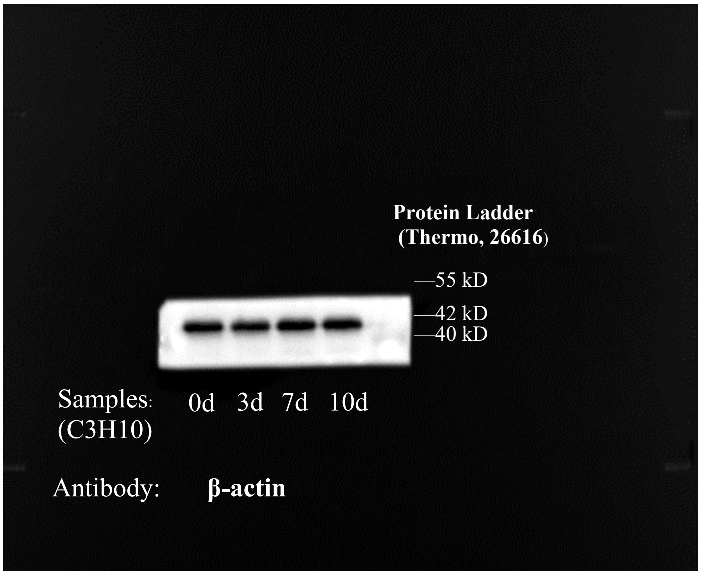

Cropped Figure 1D:

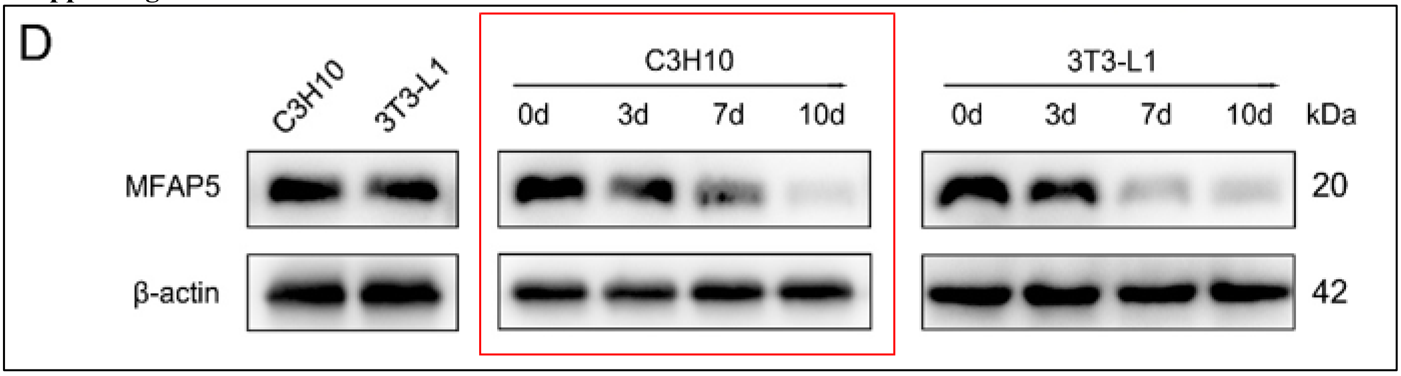

Original Images supporting Figure 1D:

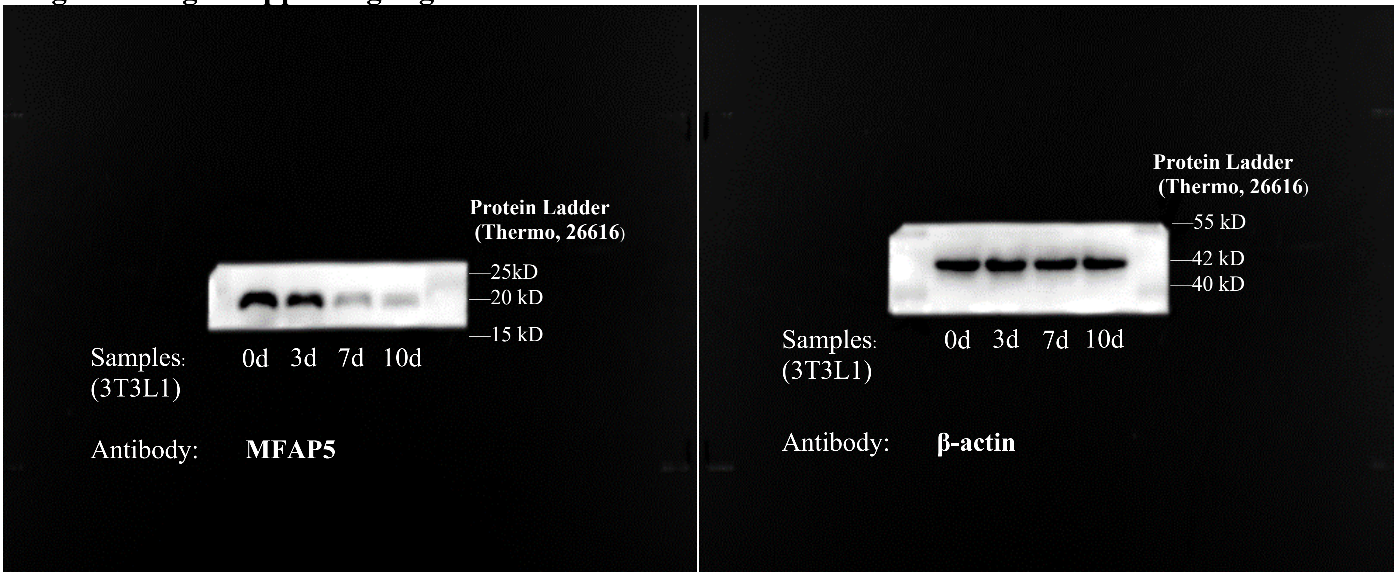

Cropped Figure 1D:

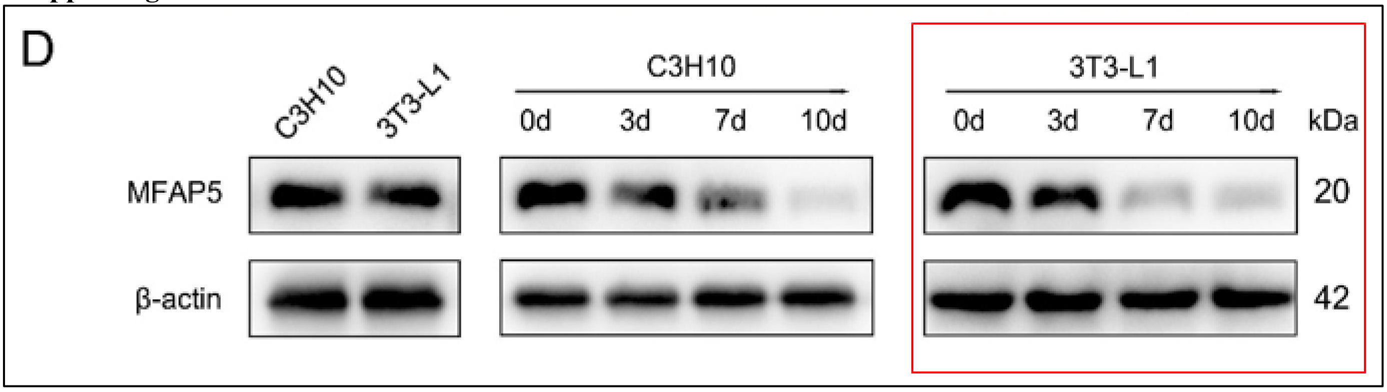

Original Images supporting Figure 2A:

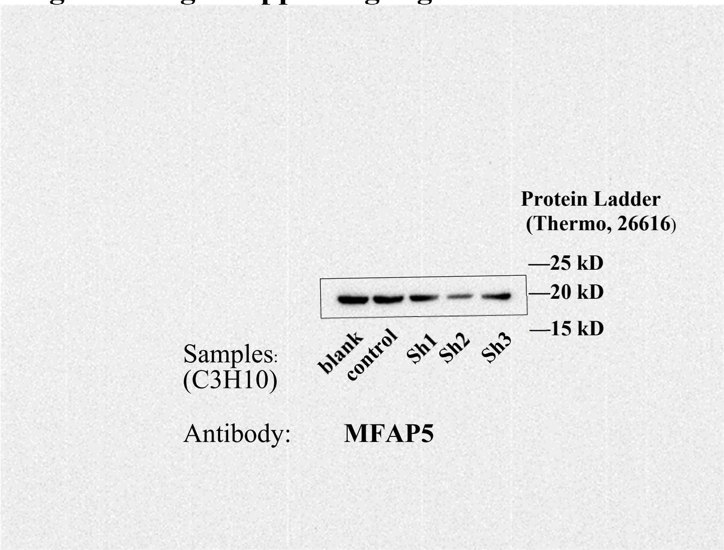

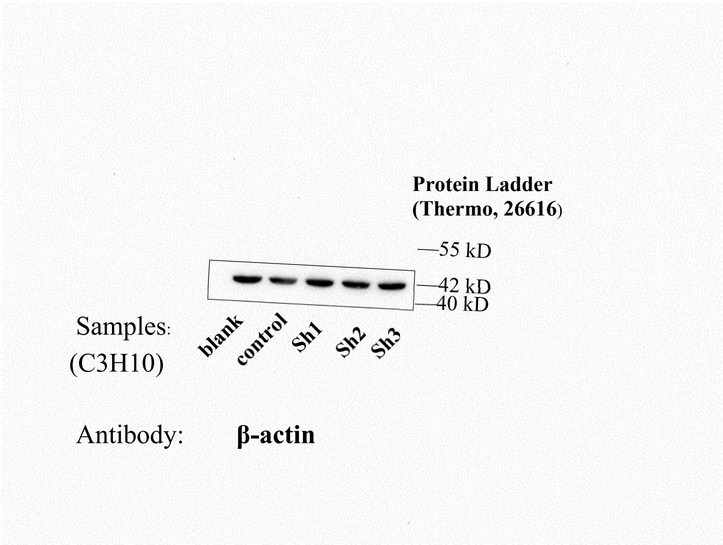

Cropped Figure 2A:

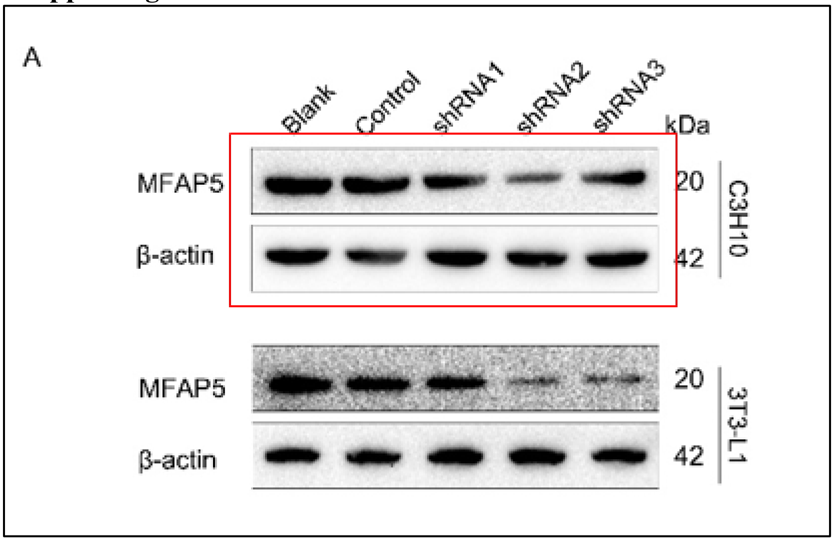

Original Images supporting Figure 2A:

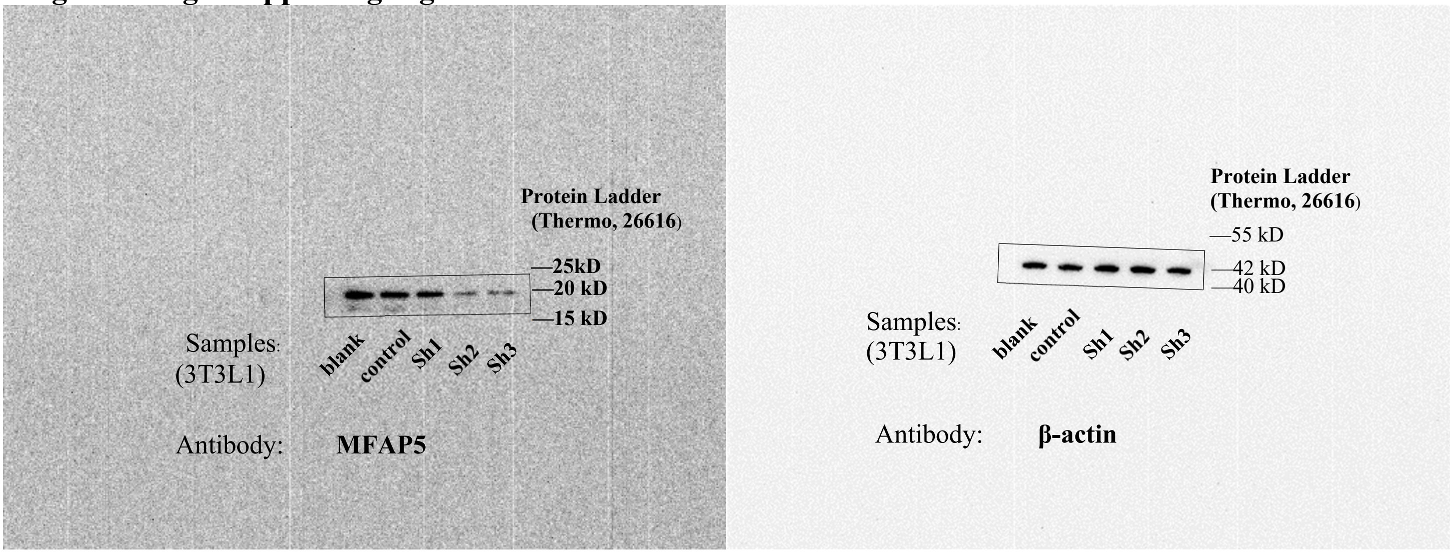

Cropped Figure 2A:

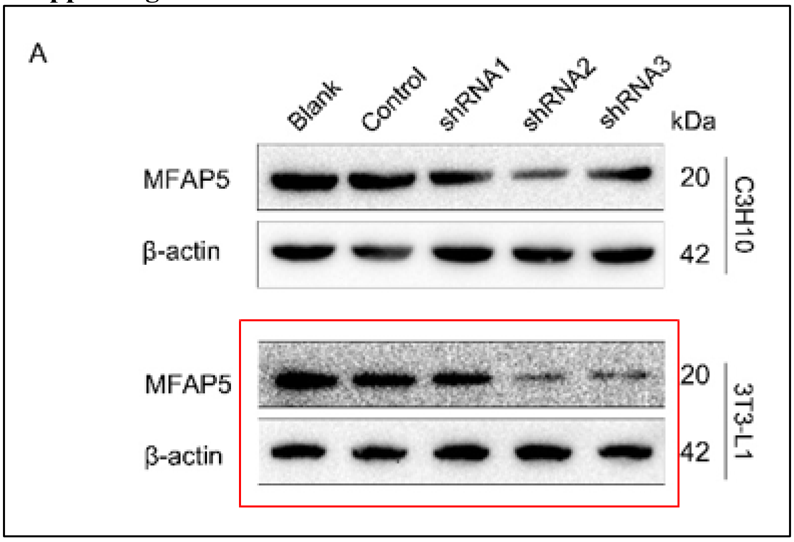

Original Images supporting Figure 3A:

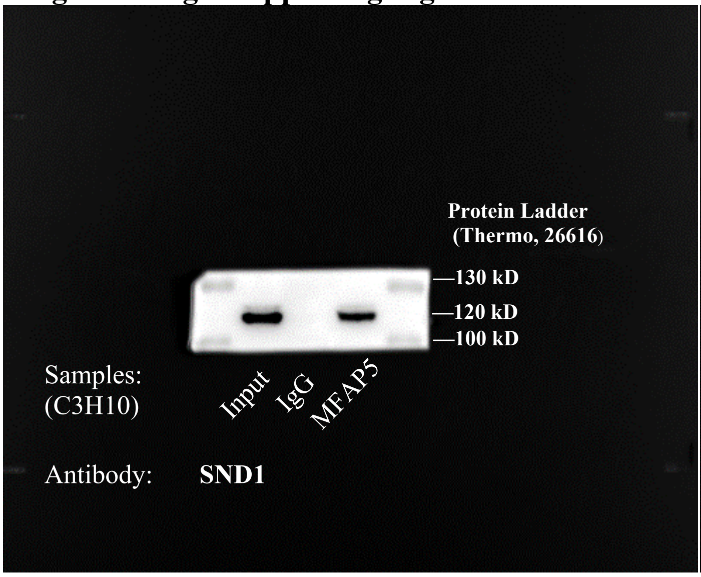

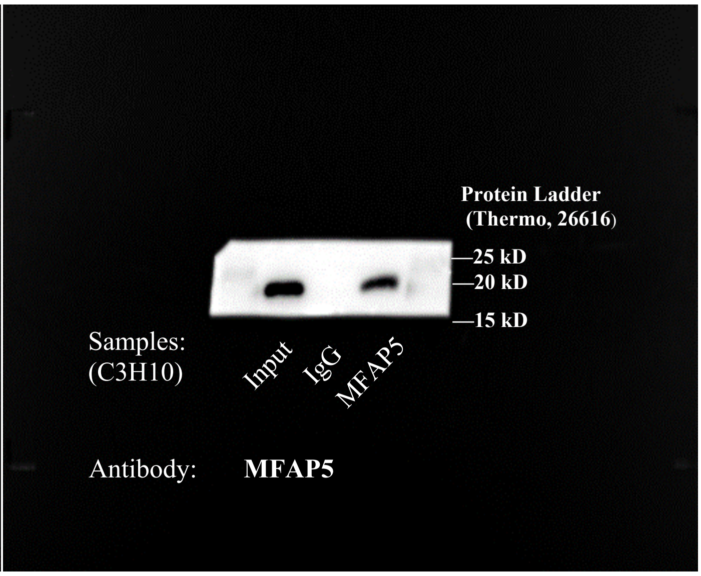

Cropped Figure 3A:

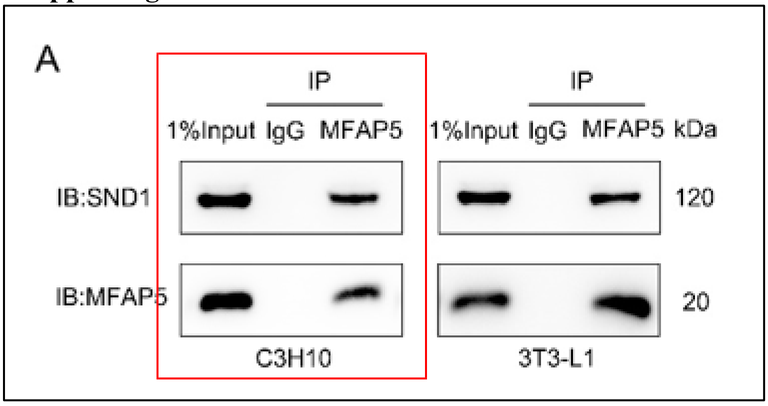

Original Images supporting Figure 3A:

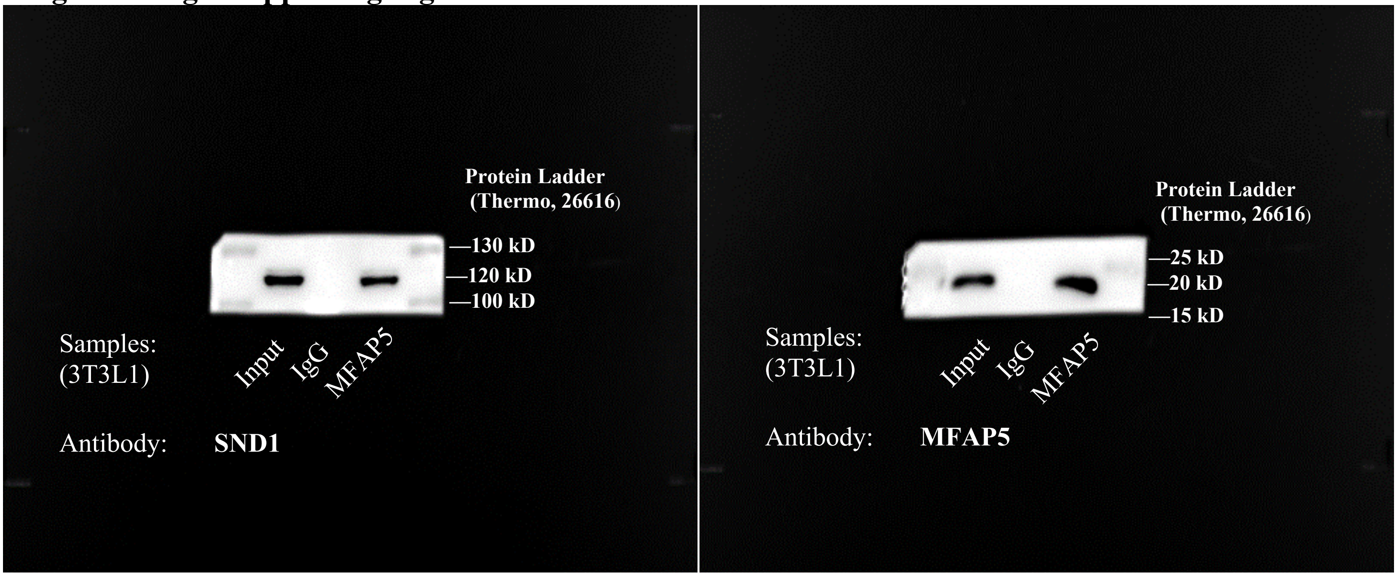

Cropped Figure 3A:

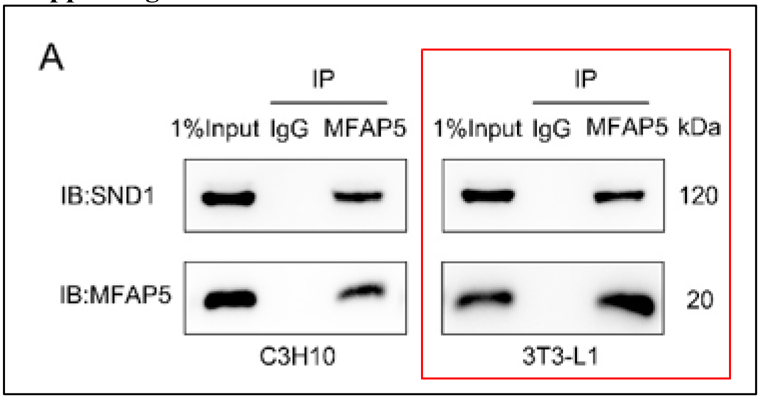

Original Images supporting Figure 3B:

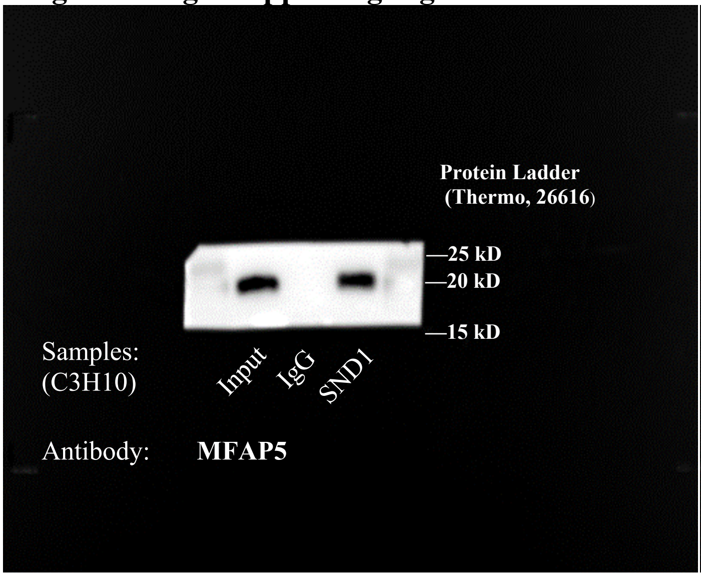

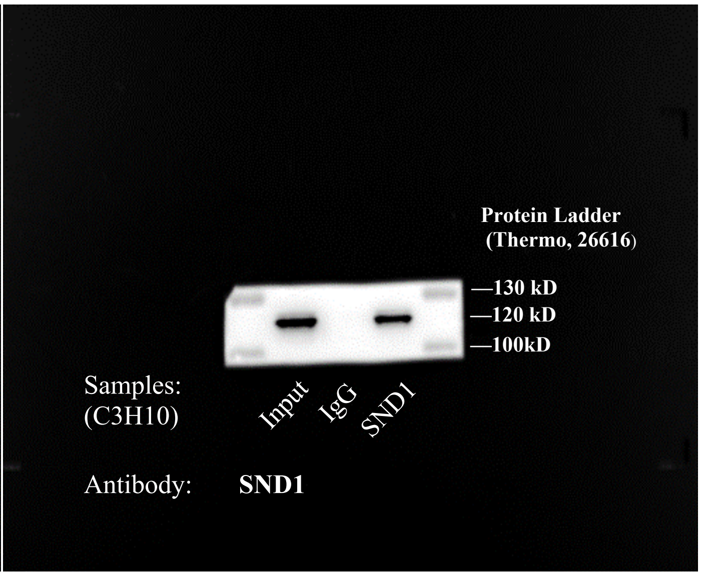

Cropped Figure 3B:

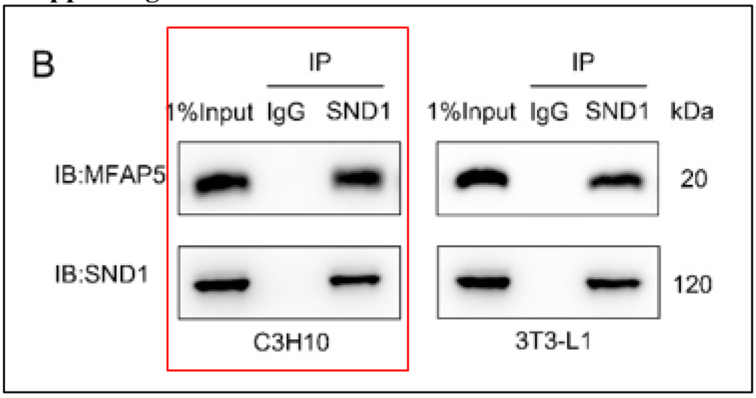

Original Images supporting Figure 3B:

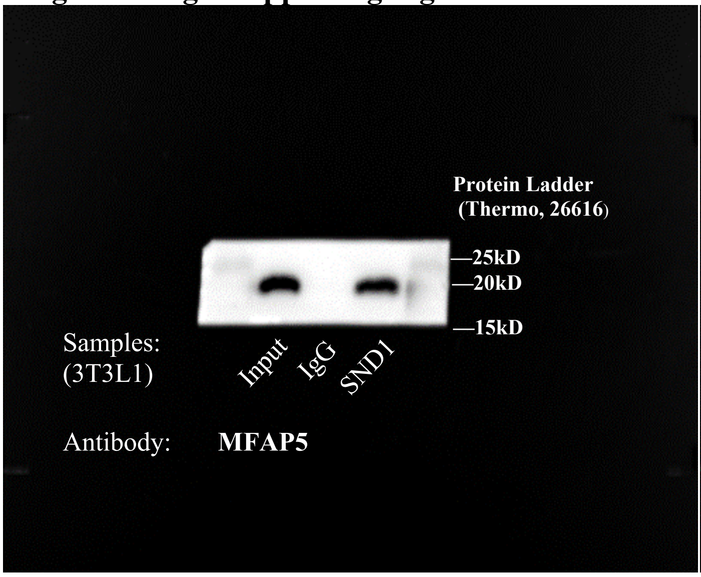

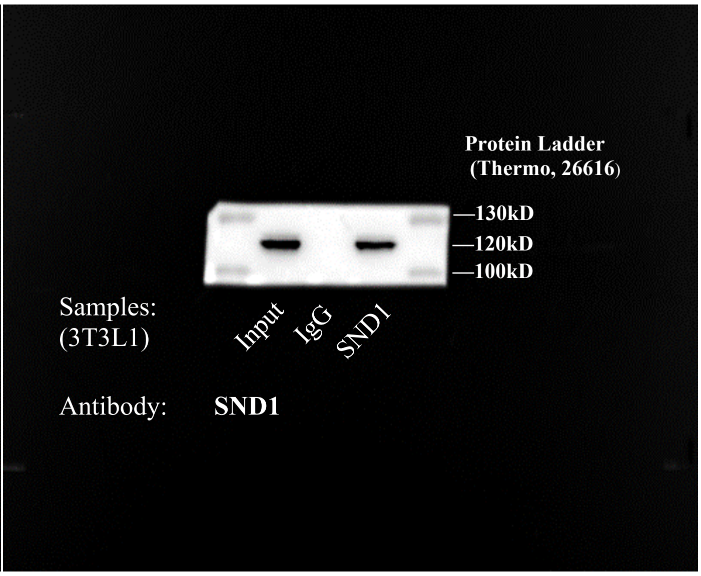

Cropped Figure 3B:

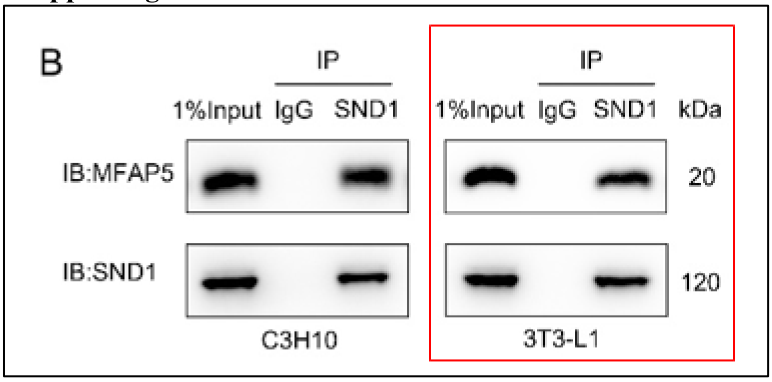

Original Images supporting Figure 3C:

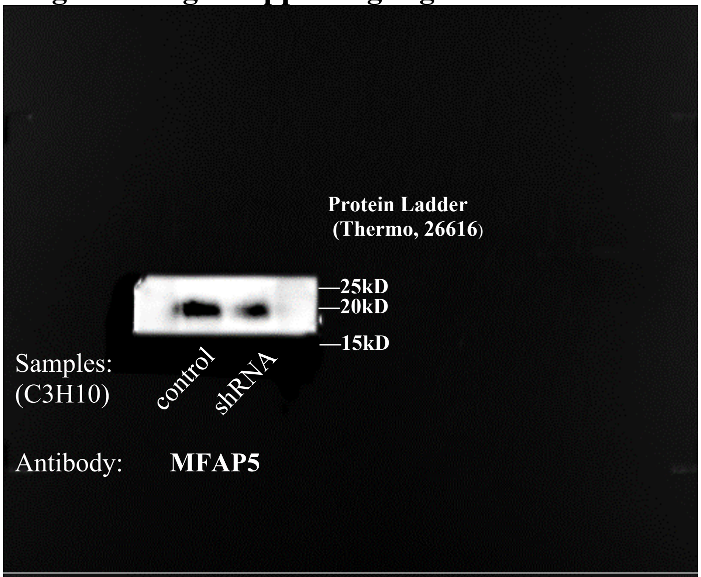

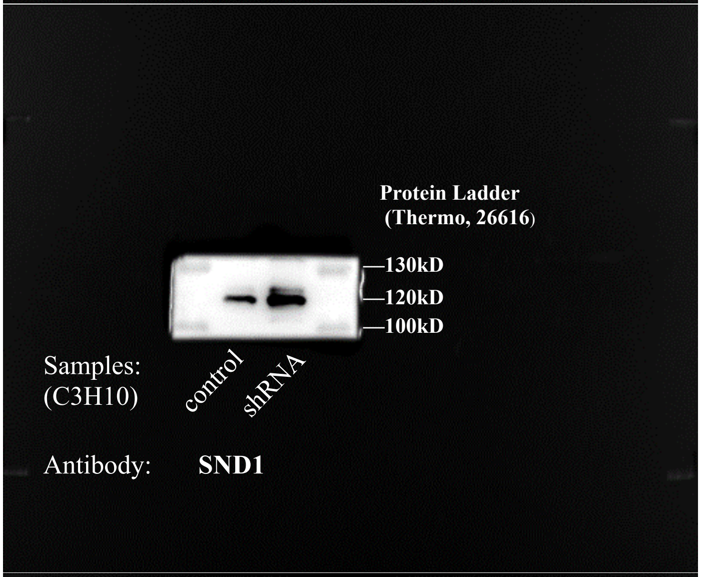

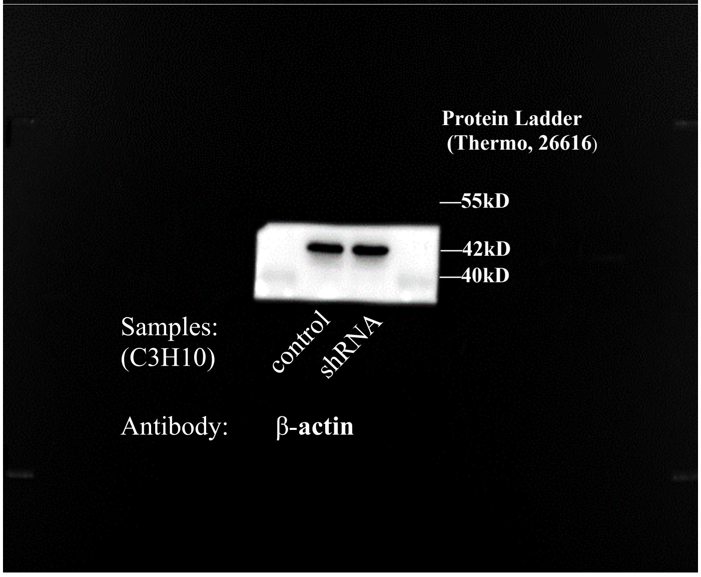

Cropped Figure 3C:

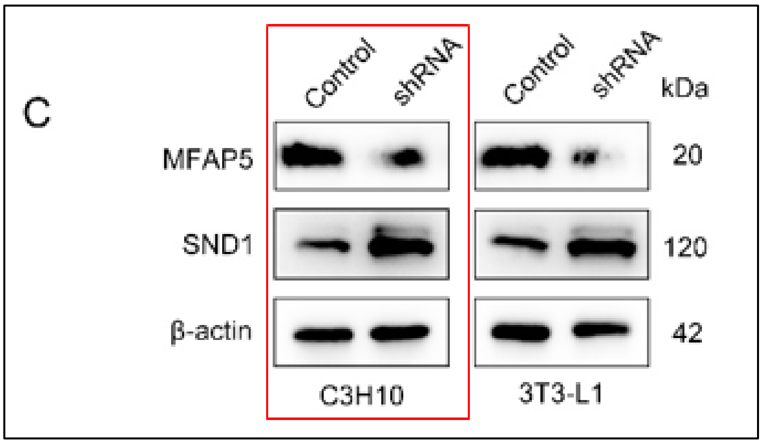

Original Images supporting Figure 3C:

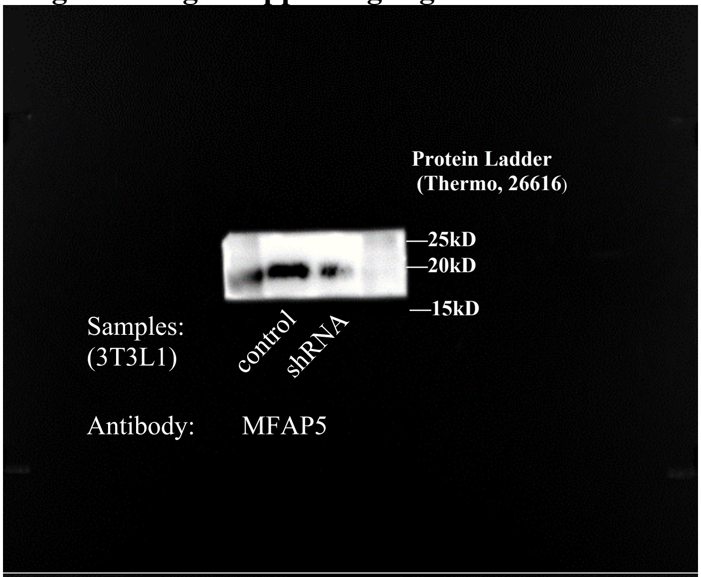

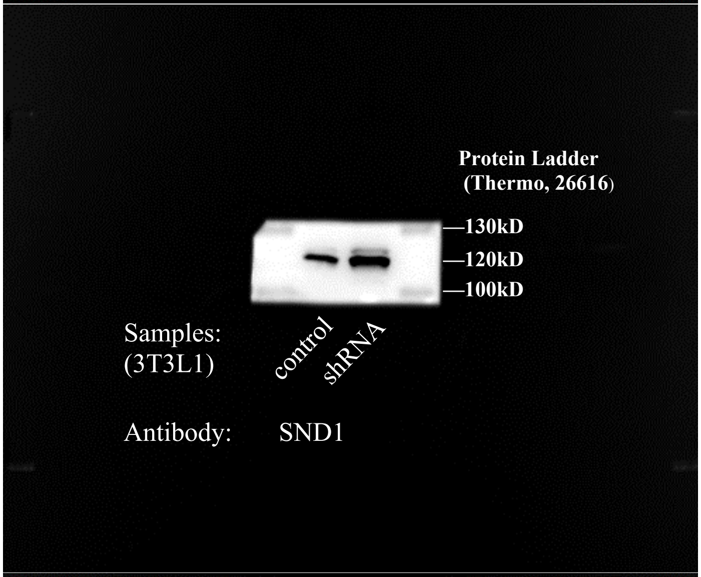

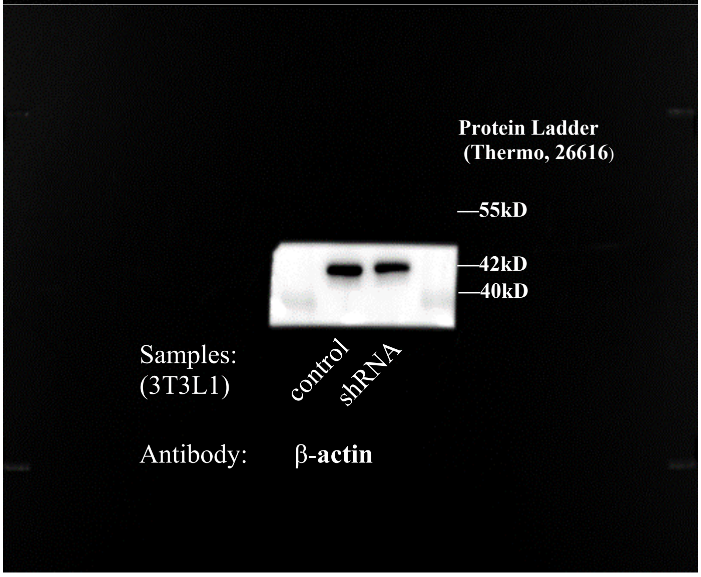

Cropped Figure 3C:

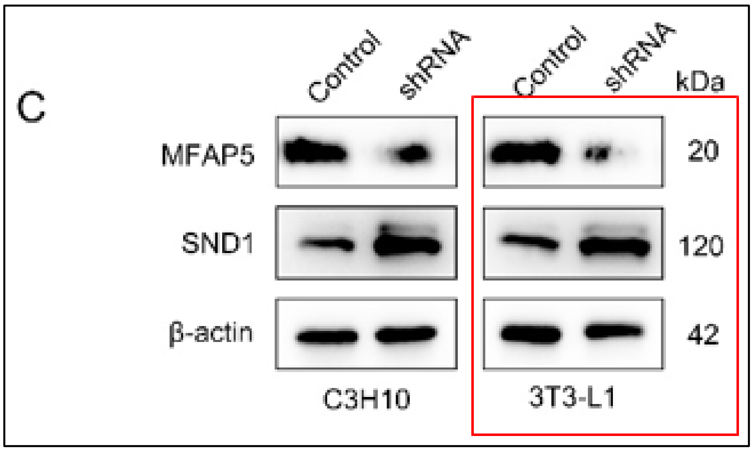

Original Images supporting Figure 3D:

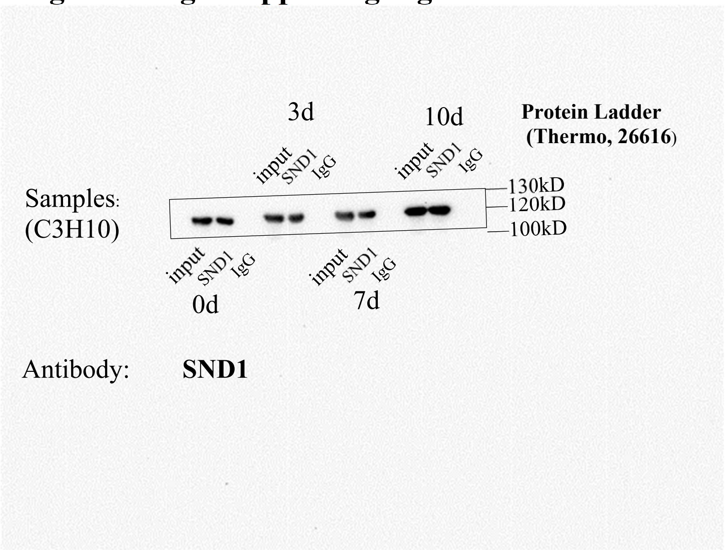

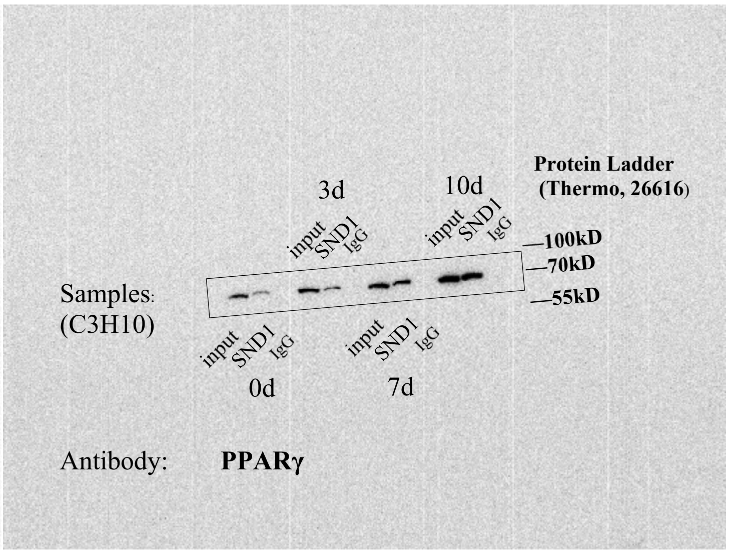

Cropped Figure 3D:

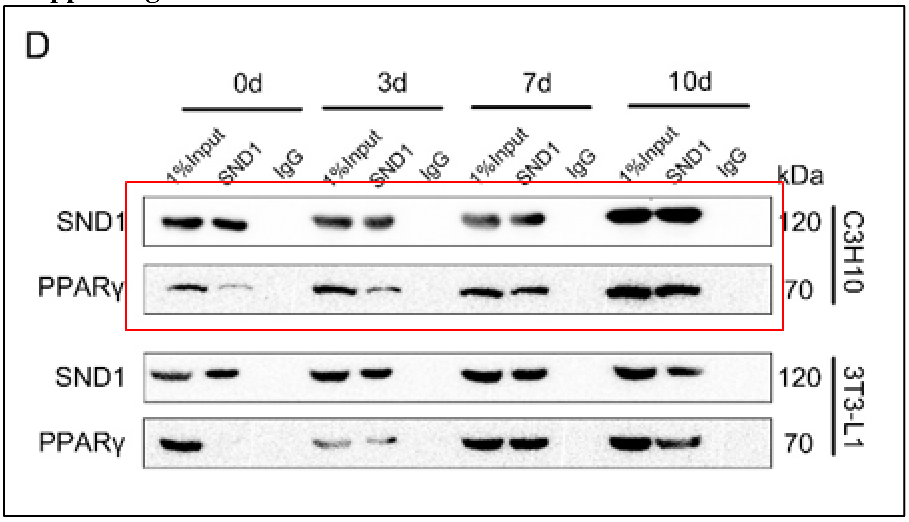

Original Images supporting Figure 3D:

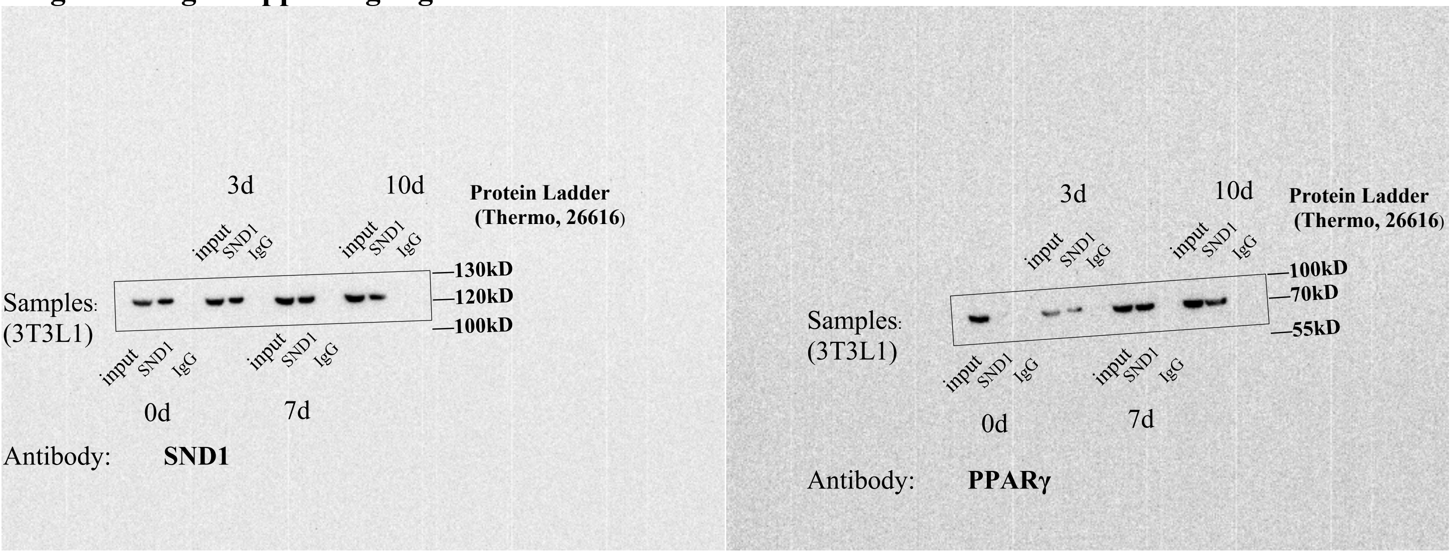

Cropped Figure 3D:

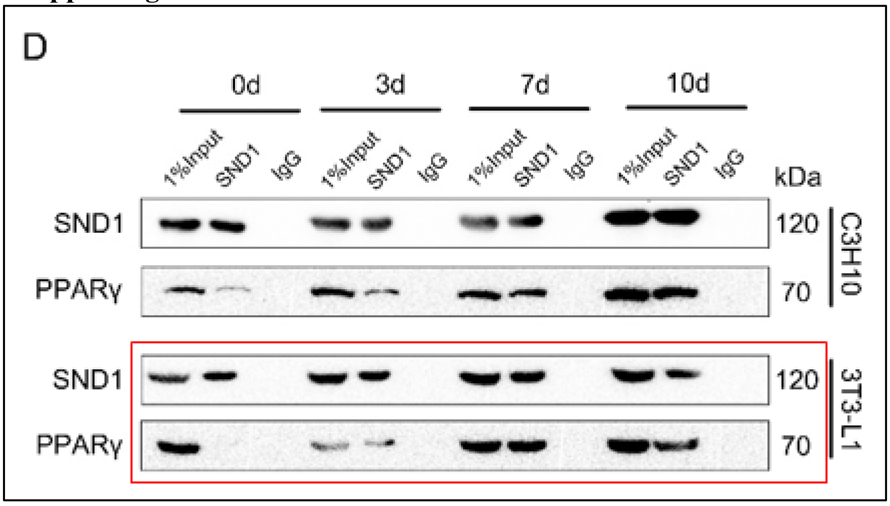

Original Images supporting Figure 3E:

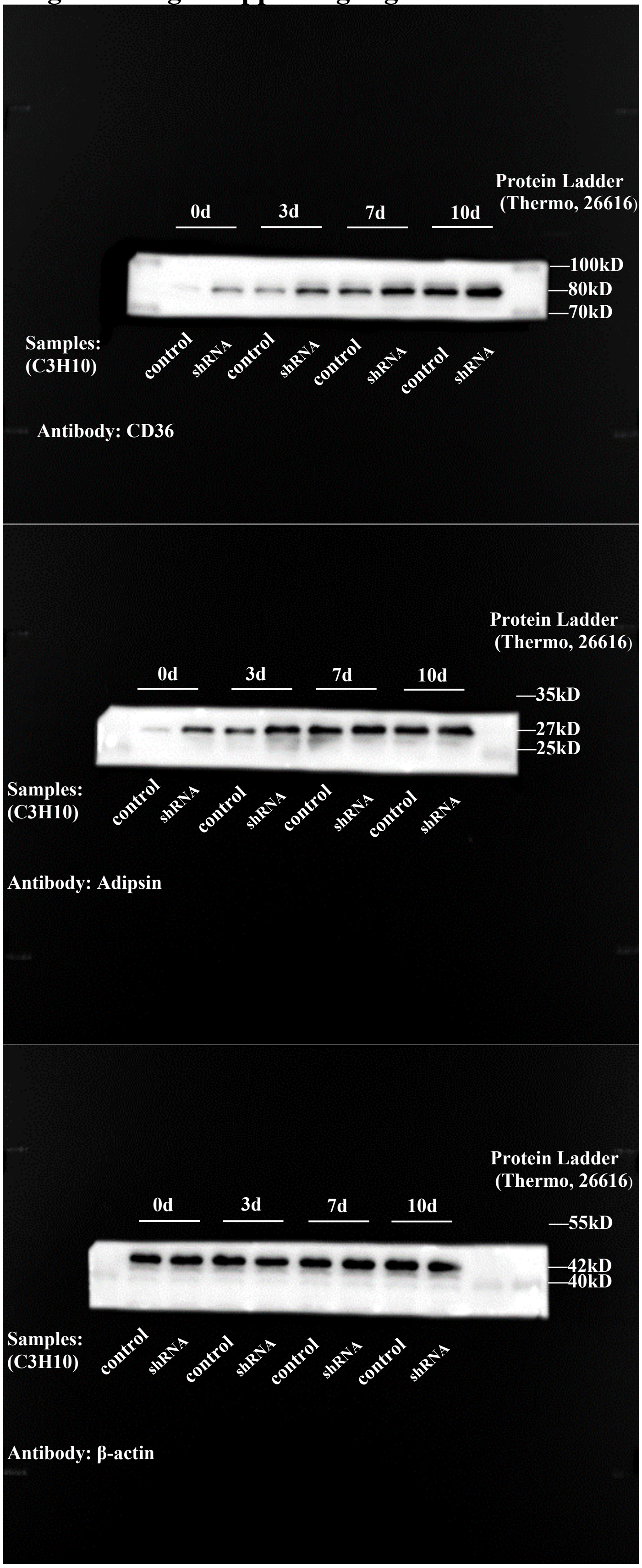

Cropped Figure 3E:

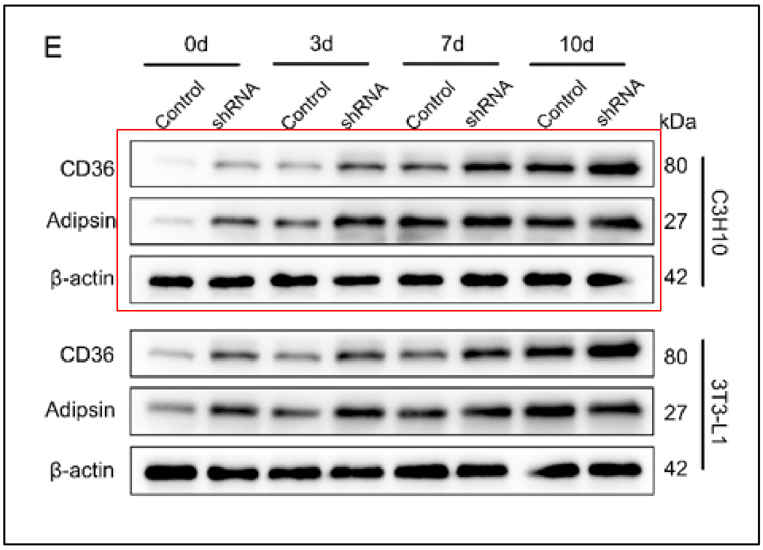

Original Images supporting Figure 3E:

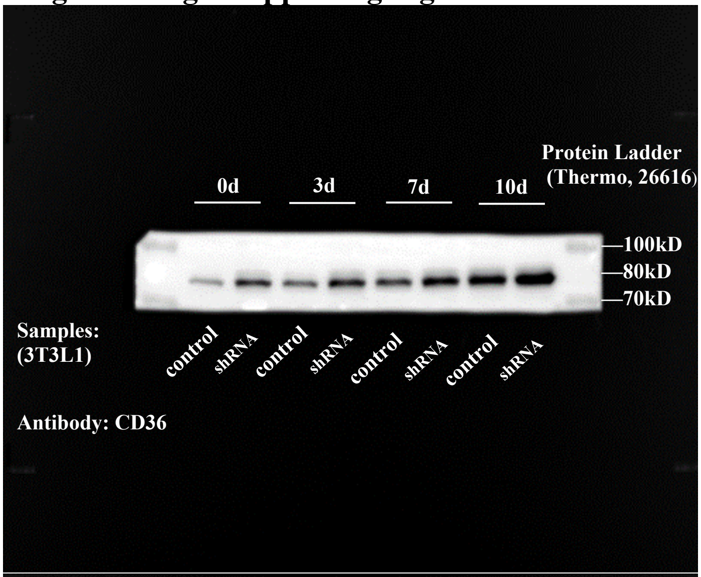

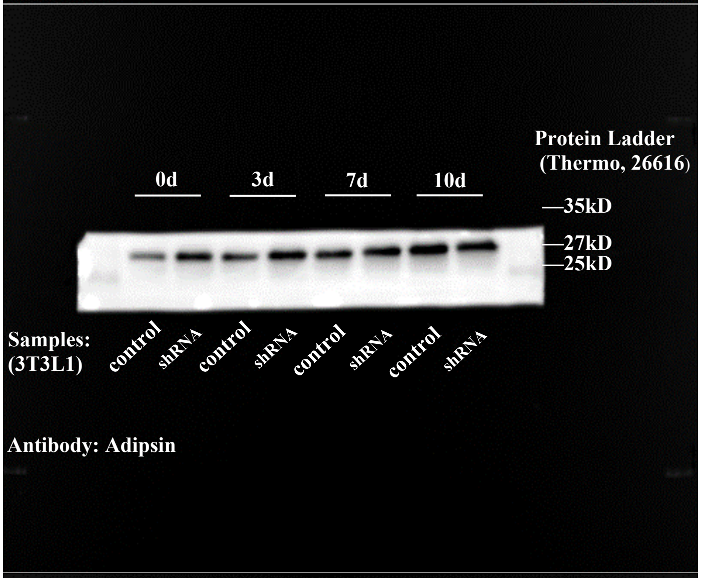

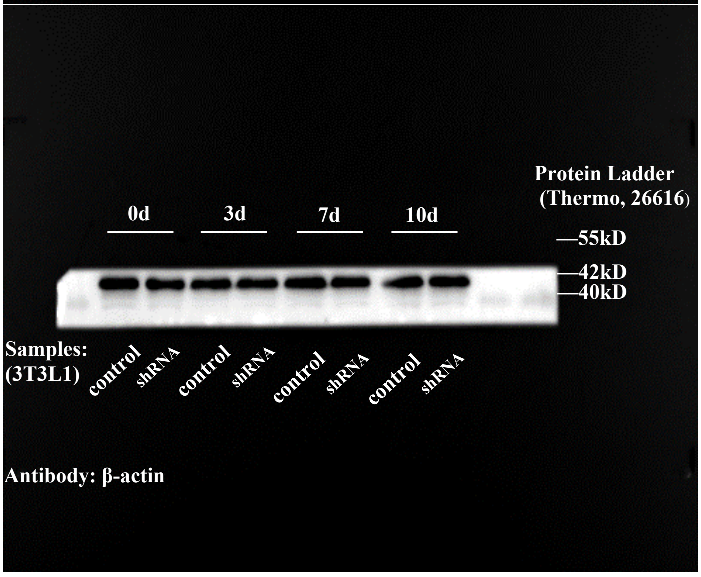

Cropped Figure 3E:

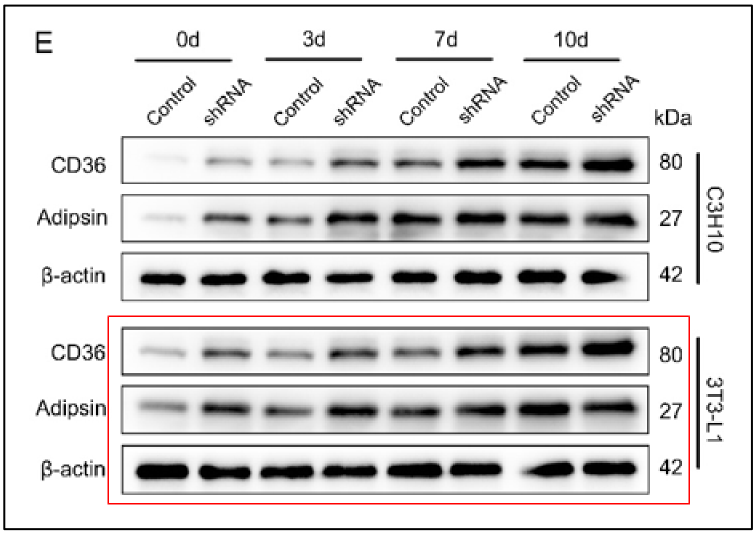

Original Images supporting Figure 4A:

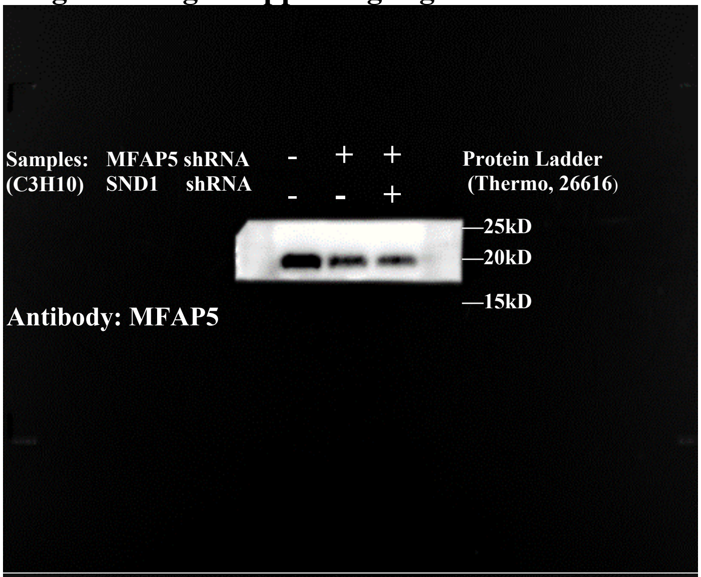

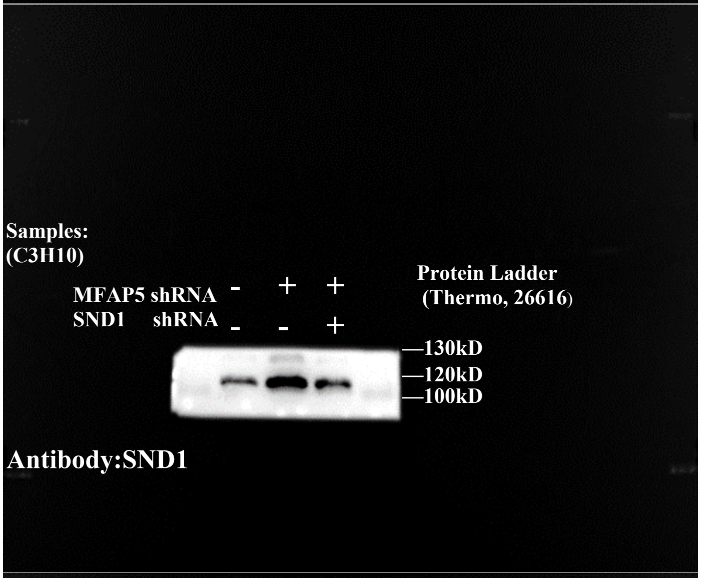

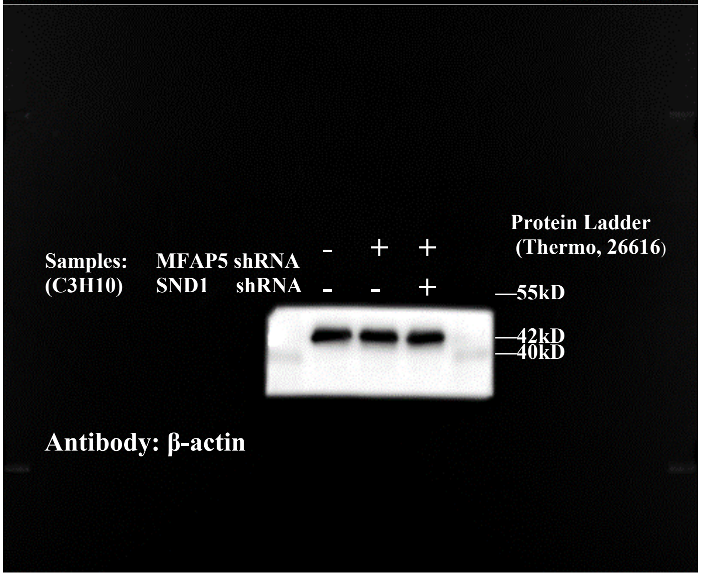

Cropped Figure 4A:

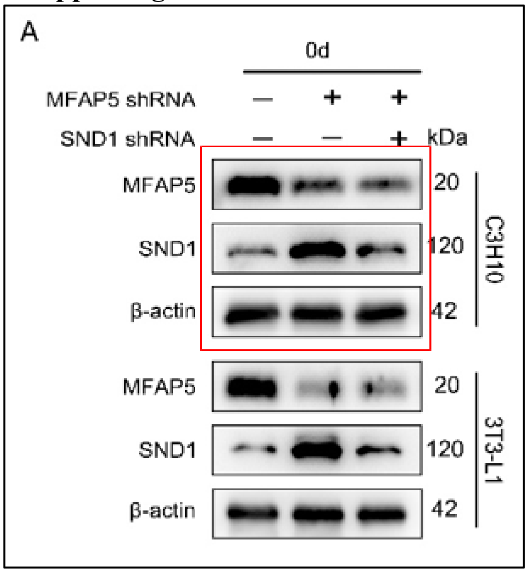

Original Images supporting Figure 4A:

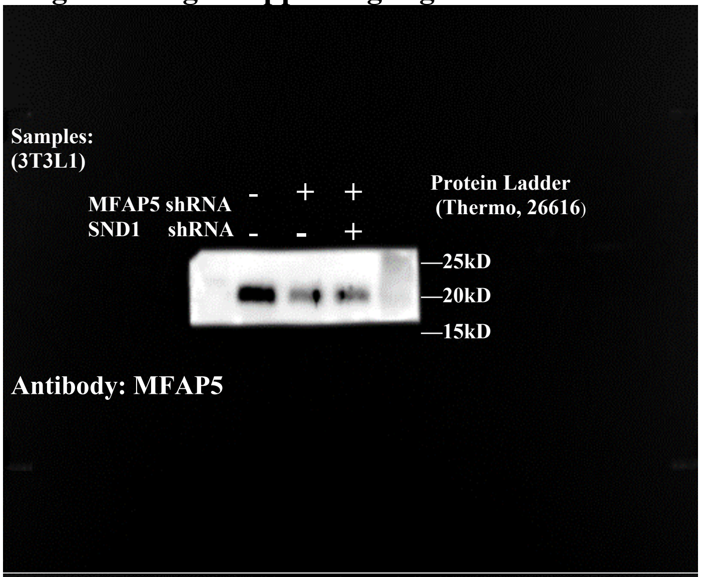

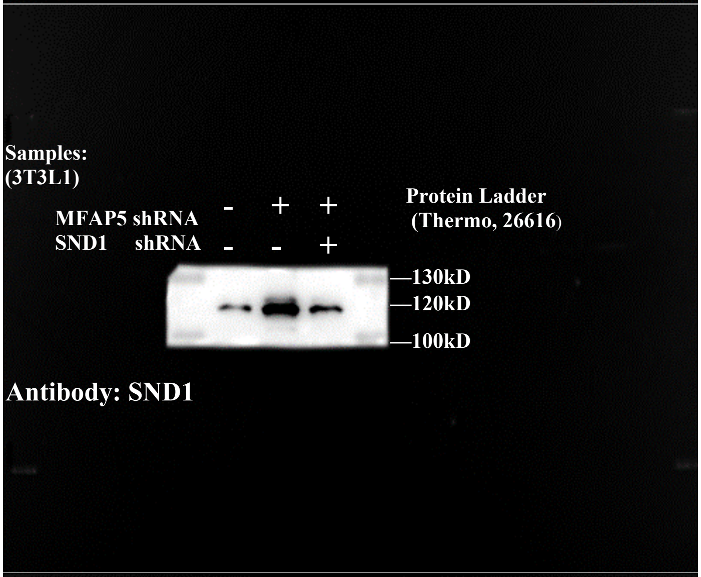

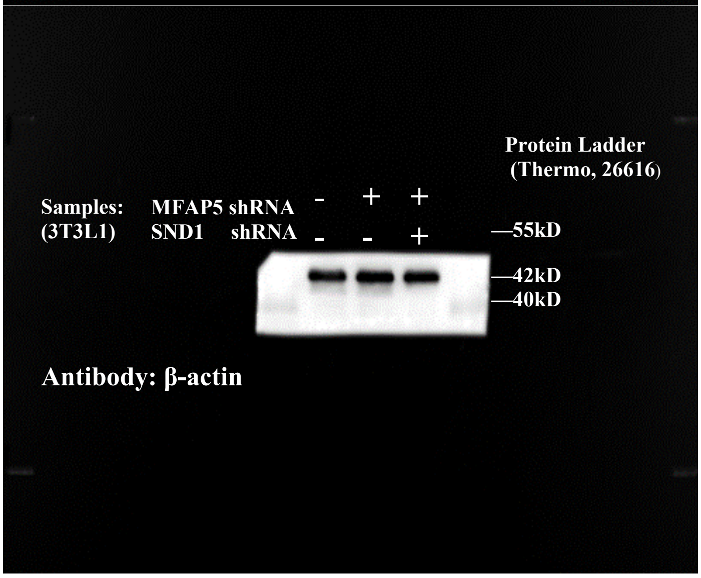

Cropped Figure 4A:

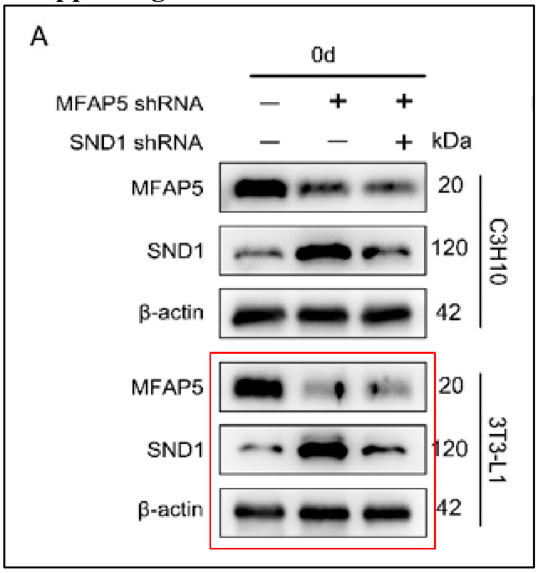

Original Images supporting Figure 4B:

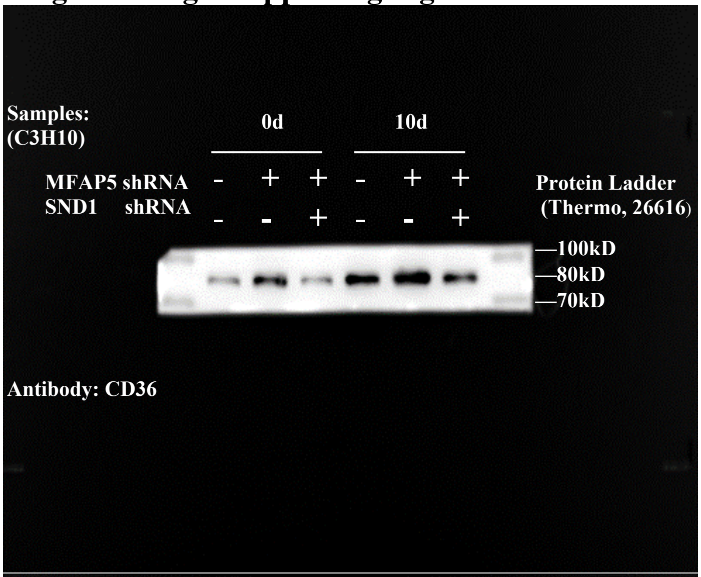

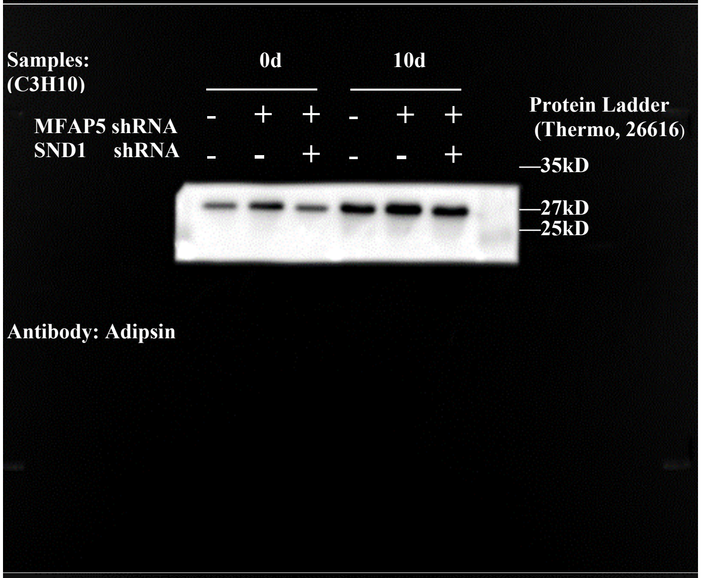

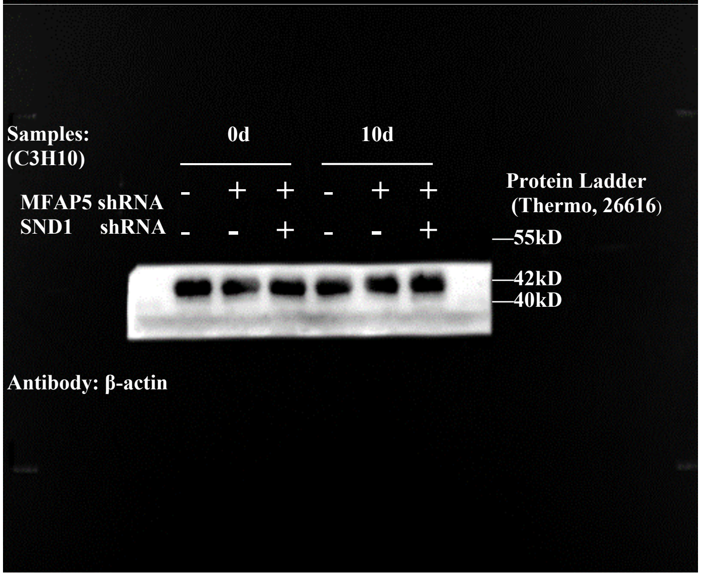

Cropped Figure 4B:

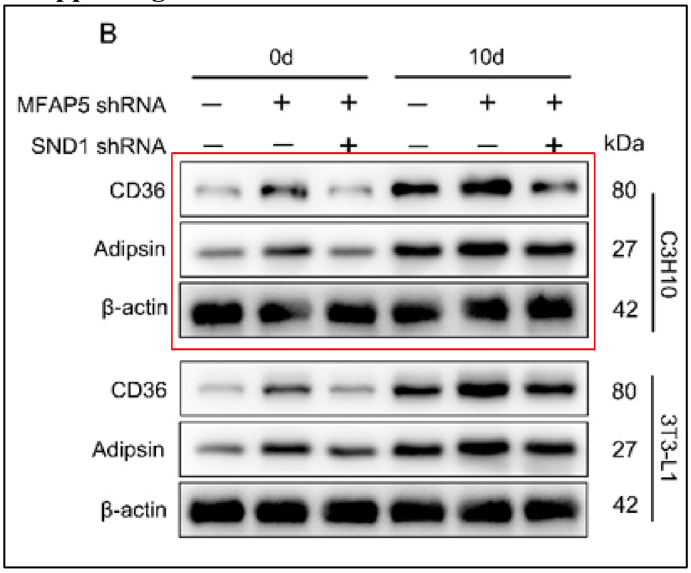

Original Images supporting Figure 4B:

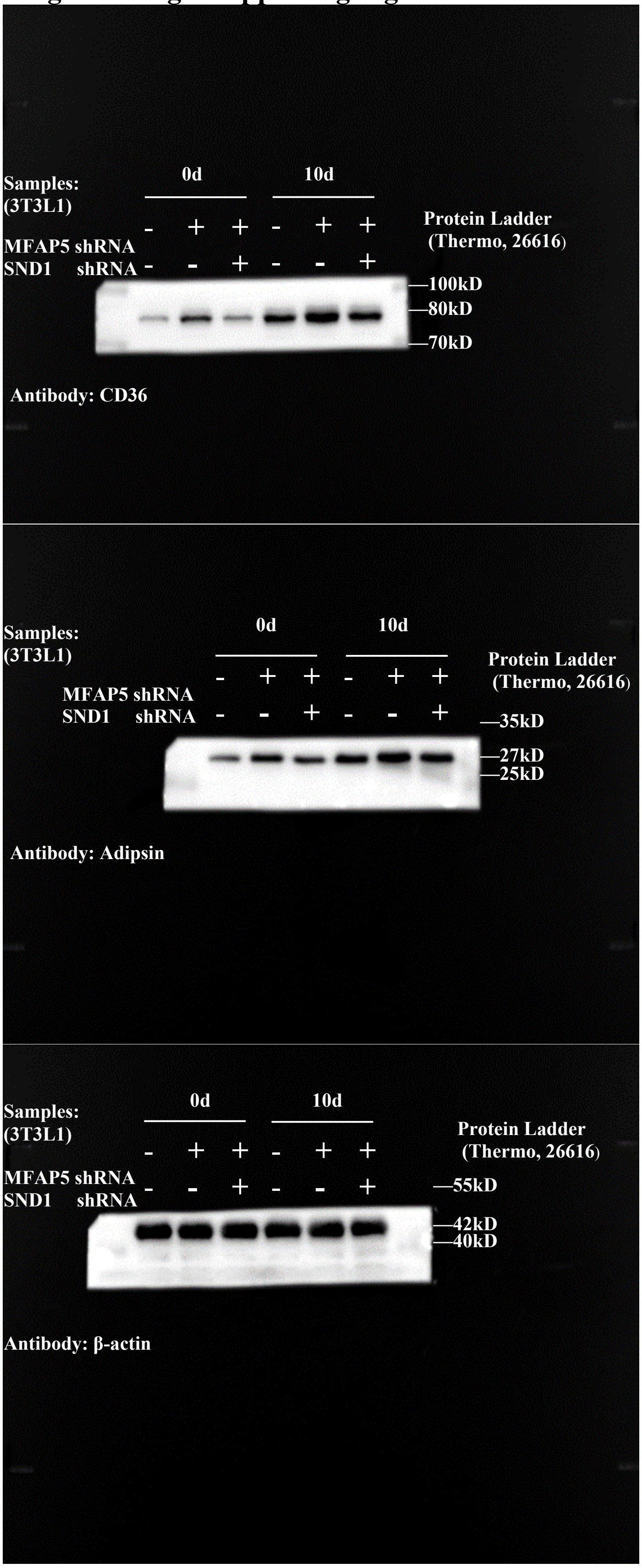

Cropped Figure 4B:

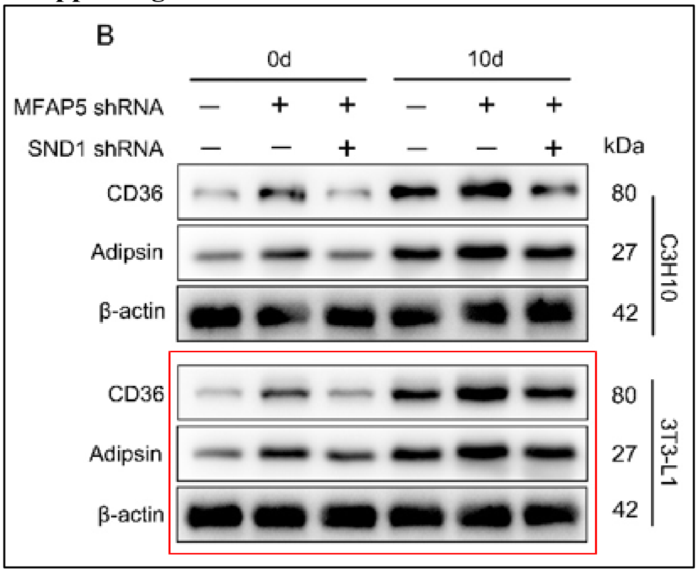

Original Images supporting Figure 5A:

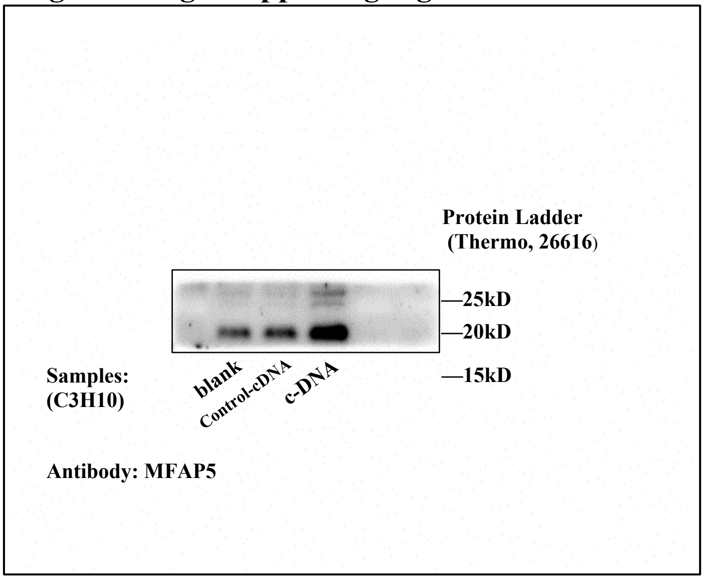

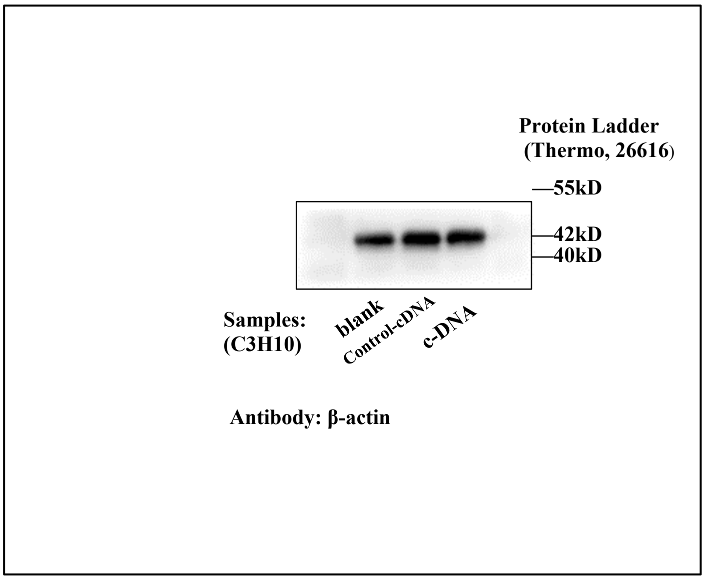

Cropped Figure 5A:

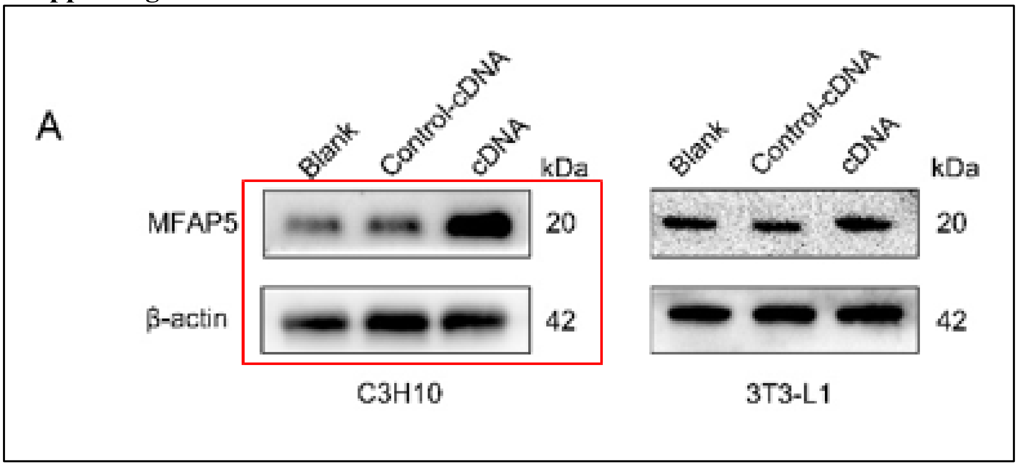

Original Images supporting Figure 5A:

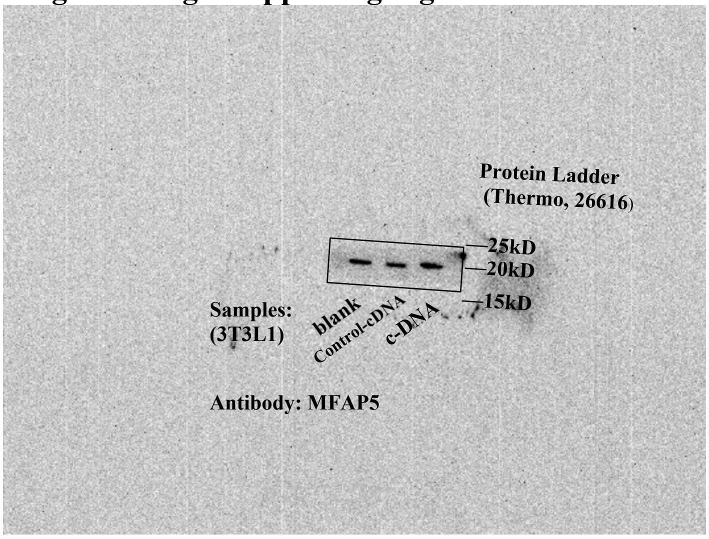

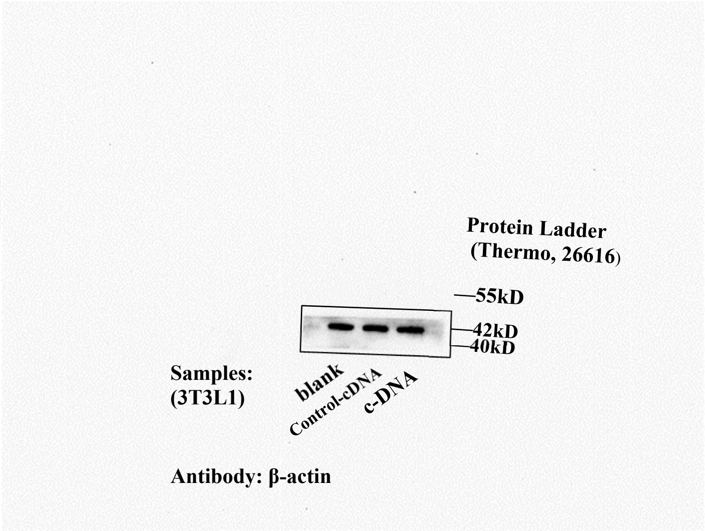

Cropped Figure 5A:

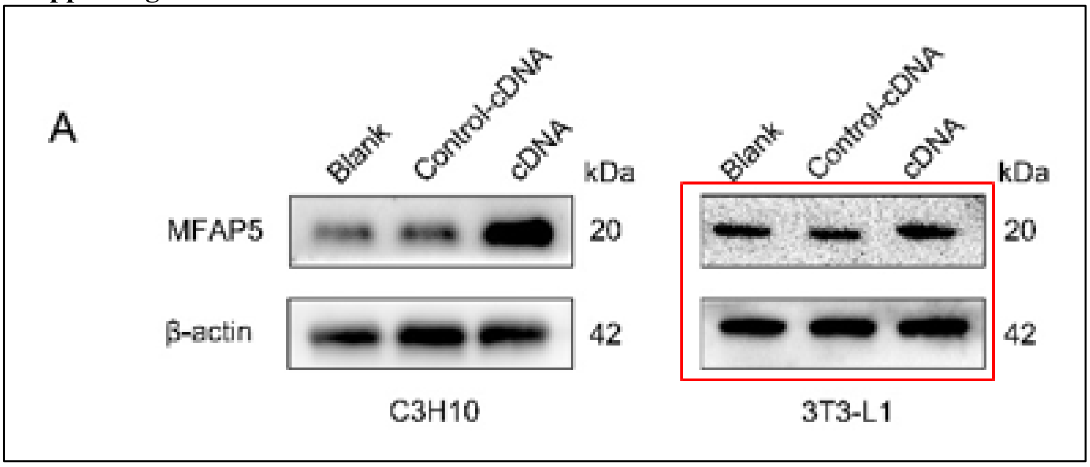

Original Images supporting Figure 5E:

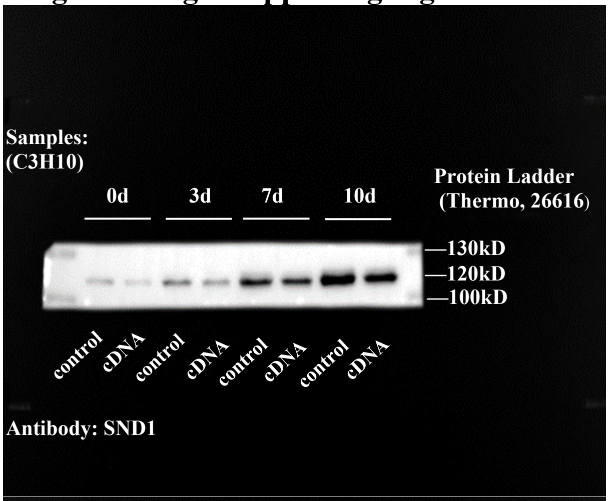

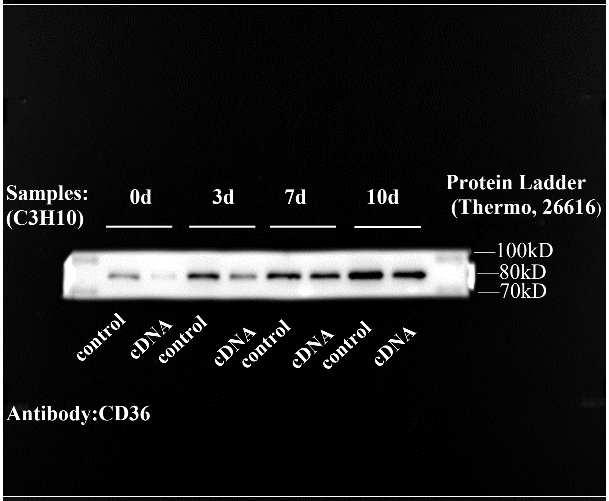

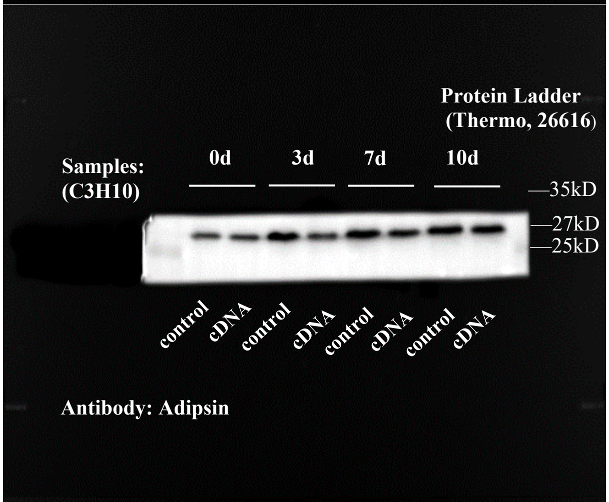

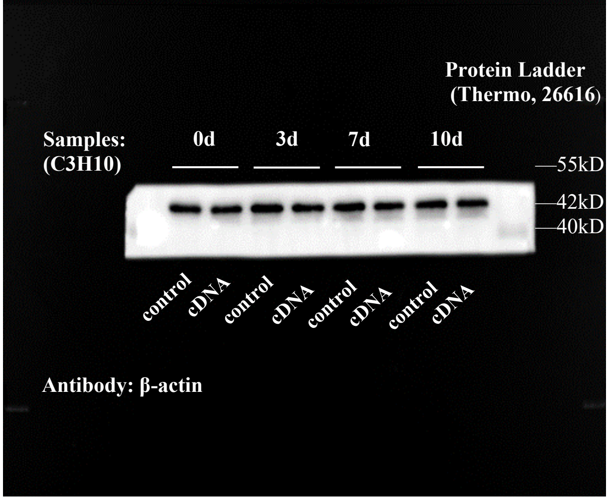

Cropped Figure 5E:

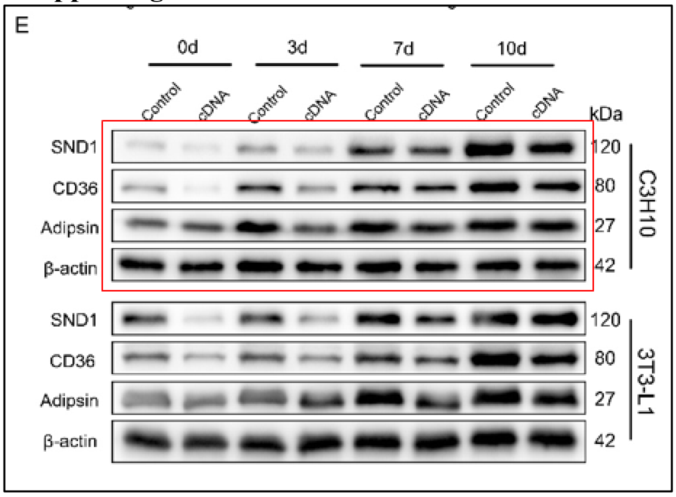

Original Images supporting Figure 5E:

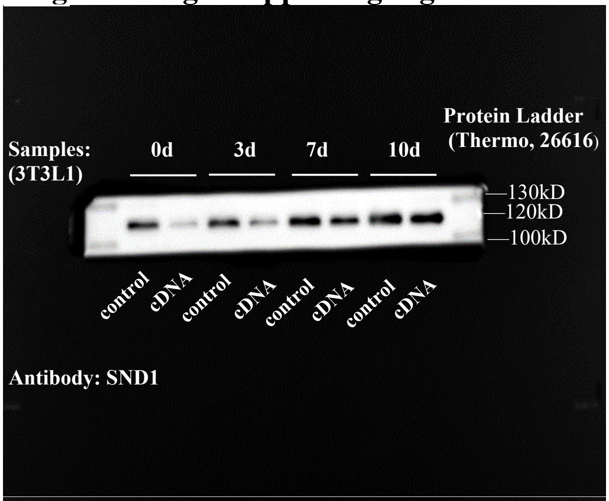

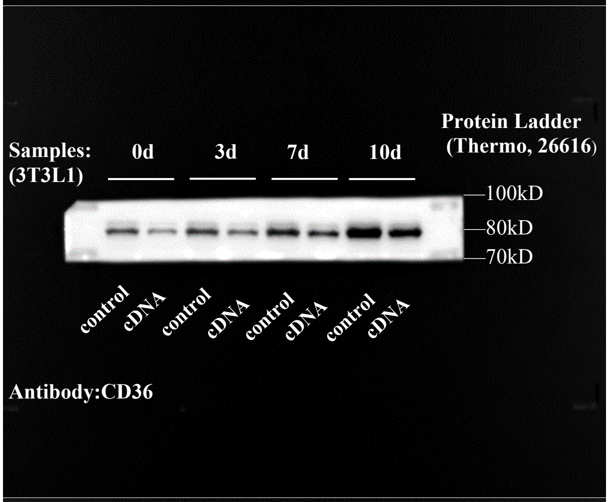

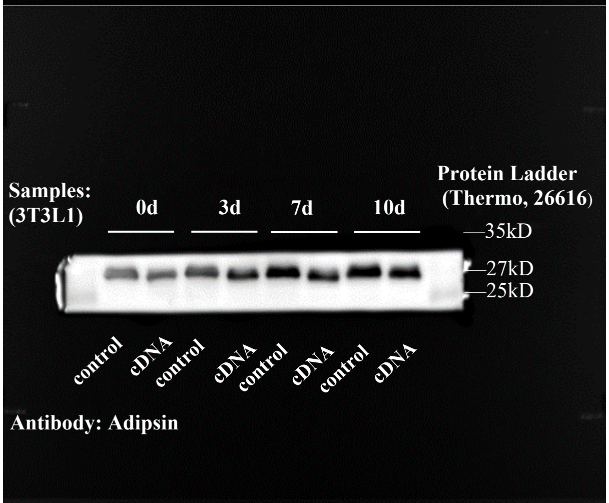

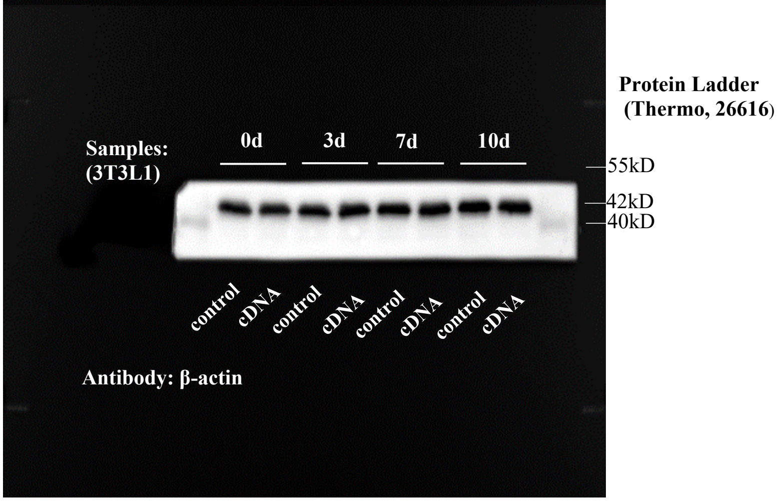

Cropped Figure 5E:

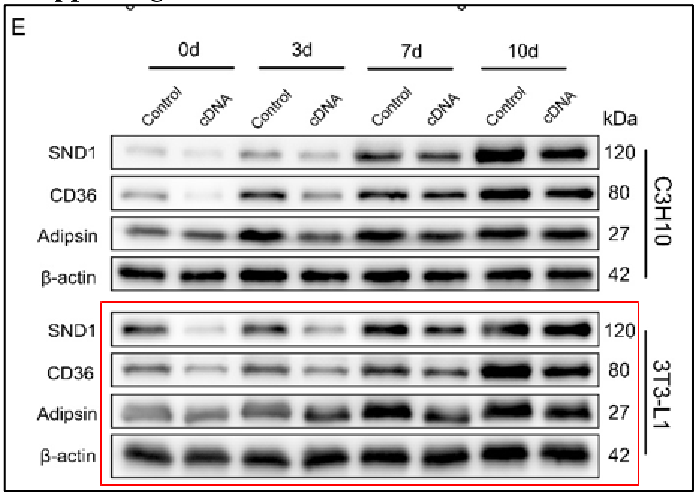

Supplement: Supplementary file 1 — Supplementary Information. [file 41598_2023_32868_MOESM1_ESM.pdf]
